# Lipid-Lowering Nutraceuticals for an Integrative Approach to Dyslipidemia

**DOI:** 10.3390/jcm12103414

**Published:** 2023-05-11

**Authors:** Brian Cheung, Geeta Sikand, Elizabeth H. Dineen, Shaista Malik, Ailin Barseghian El-Farra

**Affiliations:** 1 Susan Samueli Integrative Health Institute, 856 Health Sciences Road, Irvine, CA 92617, USA; 2 Division of Cardiology, University of California, Irvine, CA 92521, USA

**Keywords:** apolipoprotein B, atherosclerosis, cardiometabolic, cardiovascular disease, cardiovascular risk reduction, complementary medicine, integrative medicine, lipoprotein (a)

## Abstract

Dyslipidemia is a treatable risk factor for atherosclerotic cardiovascular disease that can be addressed through lifestyle changes and/or lipid-lowering therapies. Adherence to statins can be a clinical challenge in some patients due to statin-associated muscle symptoms and other side effects. There is a growing interest in integrative cardiology and nutraceuticals in the management of dyslipidemia, as some patients desire or are actively seeking a more natural approach. These agents have been used in patients with and without established atherosclerotic cardiovascular disease. We provide an updated review of the evidence on many new and emerging nutraceuticals. We describe the mechanism of action, lipid-lowering effects, and side effects of many nutraceuticals, including red yeast rice, bergamot and others.

## 1. Introduction

Atherosclerotic cardiovascular disease (ASCVD) refers to the accumulation of plaque in arteries, leading to cardiovascular disease, cerebrovascular disease, and peripheral arterial disease. The complex interplay of many factors can lead to the pathogenesis of ASCVD. Chronic inflammation is one of these important aspects and is also involved in the erosion and rupture of unstable plaque [1]. Endothelial dysfunction is another important aspect in the pathogenesis of ASCVD and is primarily mediated by nitric oxide and prostacyclins [2,3]. It can be non-invasively evaluated via flow-mediated dilation (FMD), but further research is needed to elucidate how dyslipidemia affects FMD values [3]. Diabetes mellitus type 2 (DM2) is also a risk factor associated with microvascular and macrovascular disease. It is also associated with lower high-density lipoprotein cholesterol (HDL-C) and elevated triglycerides (TG), nitrotyrosine, nitrated low-density lipoprotein-cholesterol (LDL-C), and nitrated HDL-C, which are associated with an increased risk for cardiovascular disease [4].

Dyslipidemia is an important treatable risk factor for ASCVD, and the estimated global prevalence of elevated cholesterol, according to the World Health Organization, is about 40% [5]. It is strongly associated with a lifetime exposure to elevated LDL-C in longitudinal studies [1,6,7]. Patients with a LDL-C ≥ 4.91 mmol/L (≥190 mg/dL) should be evaluated for familial hypercholesterolemia, which is frequently underdiagnosed, as about 85% of those with the autosomal dominant genetic disorder are unaware of their diagnosis [8]. Other derangements in the lipid profile may be considered risk-enhancing factors, which may favor initiation or intensification of therapy in certain patients. Such factors include persistently elevated TG of ≥2.0 mmol/L (≥175 mg/dL), apolipoprotein B (apoB) ≥ 130 mg/dL, or lipoprotein (a) (Lp(a)) ≥ 50 mg/dL or 125 nmol/L [1]. 

Statins, which are 3-hydroxy-3-methylglutaryl-coenzyme A (HMG-CoA) reductase inhibitors, are the cornerstone in the management of dyslipidemia and are frequently first-line therapeutics [9]. Robust evidence demonstrates that statins, ezetimibe, and proprotein convertase subtilisin/kexin type 9 (PCSK9) inhibitors substantially reduce ASCVD risk [6,7,9,10]. 

However, adherence to statin therapy can be a clinical challenge because some patients can develop side effects, such as statin-associated muscle symptoms (SAMS) [11]. Some degree of statin intolerance is reported in 5–30% of patients and contributes to reduced statin adherence [10]. Furthermore, some patients are hesitant to starting a statin due to concern for potential side effects. 

More patients are actively seeking integrative therapies, including nutraceuticals, from clinicians with expertise in integrative cardiology [12]. The estimated global prevalence of dietary supplement and nutraceutical use in patients with cardiovascular disease is 36% [13]. Nutraceuticals can be considered in the management of dyslipidemia for their lipid-lowering effects in patients who are not on statins or other LDL-lowering therapies due to a desire for a more natural approach. They can also be considered as adjunctive treatments to conventional LDL-lowering therapies that have not achieved lipid thresholds. Nutraceuticals can also be considered in patients with SAMS or other side effects, although they are not without their own side effects. Thus, clinicians have a need for high-quality evidence-based recommendations relating to the use of nutraceuticals [14,15].

Due to the growing interest in the field of integrative cardiology, many new clinical trials, systemic reviews, and meta-analyses have recently been published on nutraceuticals. We review the updated literature on novel and emerging nutraceuticals used in the management of dyslipidemia and their lipid-lowering effects. 

## 2. Methods

A literature review was performed searching Pubmed, Scopus, and Google Scholar. A search for the most recent published meta-analysis, systemic reviews, and randomized control trials (RCT) was performed for each nutraceutical. Publications not written in English or lacking accompanying English translations were excluded. 

The level of evidence and strength of recommendations for particular lipid-lowering therapies were evaluated and graded according to predefined scales in the legend of Table 1, which also shows a summary of LDL-lowering effects of nutraceuticals [16]. 

## 3. Nutraceuticals and Their Main Effects on LDL-C

### 3.1. Red Yeast Rice Extract

Red yeast rice (RYR) extract is a traditional Chinese supplement that is produced via fermenting yeast (*Monascus purpureus*) grown on white rice and contains sterols, isoflavones, and polyketides such as monacolin K [14,15,16,126]. The lactone form of monacolin K is structurally identical to lovastatin and requires conversion into the active hydroxyl acid form via the small intestine and liver [16,126]. RYR extract consumption results in the reduction of circulating atherogenic LDL-C particles, which is associated with a substantial reduction in the risk of cardiovascular events. Thus, RYR extract formulations may have a role in preventing cardiovascular disease. Besides monacolin K, RYR extract contains a wide (and variable) range of chemical constituents and several monacolins (M, L, J, and X) that may also contribute in varying degrees to the lipid-lowering effect [14]. Monacolin K is an HMG-CoA reductase inhibitor and increases clearance of serum LDL-C [124]. The typical dosage of RYR extract ranges from 1.2 to 2.4 g daily. This is equivalent to about 4.8 to 10 mg of monacolin K, depending on the formulation [16]. The International Lipid Expert Panel (ILEP) recommends a daily dose of <3 mg monacolins [14].

The expected LDL-C reduction is about −15% to −25% [16]. In a recent meta-analysis of 30 RCTs, 5440 participants were treated with RYR extract compared to placebo. Those in the RYR extract arm experienced a reduction in total cholesterol (TC) (−0.74 mmol/L; 95% confidence interval [CI], −0.71 to −0.22), TG (−0.45 mmol/L; 95% CI, −0.70 to −0.21), LDL-C (−0.42 mmol/L; 95% CI, −0.78 to −0.06) and an increase in HDL-C (0.14 mmol/L; 95% CI, 0.09 to 0.20) [150]. In another meta-analysis of 15 RCTs with 1012 participants, RYR extract lowered apoB (−5.45 × 10^−4^ mmol/L; 95% CI, −6.90 × 10^−4^ to −4.00 × 10^−4^) compared to placebo [151].

Another meta-analysis investigated RYR extract’s impact on cardiovascular outcomes, and it included a total of seven RCTs in China with 10,699 participants with dyslipidemia who were not on statins. The authors found that RYR extract reduces the risk of a non-fatal myocardial infarction (MI) (relative risk [RR]  =  0.42; 95% CI, 0.34 to 0.52), revascularization (percutaneous coronary intervention and coronary artery bypass graft) (RR  =  0.58; 95% CI, 0.48 to 0.71), and sudden death (RR  =  0.71; 95% CI, 0.53 to 0.94) when compared to placebo. However, there was no significant difference in fatal MI [152].

Some of the more common adverse side effects can include gastrointestinal discomfort, rashes, and allergic reactions [125,151,152]. The prevalence of increased liver transaminases or muscle symptoms in RYR extract is comparable to that of statins [14,151,152]. Although citrinin is a nephrotoxic and hepatotoxic metabolite produced during the fermentation of RYR, a meta-analysis of 53 RCTs with 8535 participants did not demonstrate significant kidney impairment or elevated transaminases with RYR extract [14,126,151,152]. There are rare reports of rhabdomyolysis in patients with a prior history of rhabdomyolysis or polypharmacy use with RYR extract, statins, and antidepressants [126]. However, multiple meta-analyses of RCTs have shown no serious adverse reactions from RYR extract requiring hospitalizations when compared to statins and placebo [14,151,152].

Although the United States (US) Food and Drug Administration (FDA) has released a statement that statins may be considered in certain pregnant patients, such as those with homozygous familial hypercholesterolemia, special precautions should be taken with the use of RYR extract during pregnancy [125]. Citrinin-free formulations should be used, as citrinin is teratogenic [16]. The IELP recommends a minimum dose of citrinin. The no-observed-adverse-effect level is ≤0.2 µg/kg body weight per day, which is ≤14 µg/day for a 70 kg person [14]. It is unknown if RYR extract is safe during breastfeeding. Special precautions should also be taken in patients with hepatobiliary disorders [126]. Other potential interactions include those with food, CYPP450 inducers or inhibitors, antifungals, macrolides, cyclosporine, fibrates, niacin, nefazodone, protease inhibitors, statins, and verapamil [16,126].

Formulations containing RYR extract, however, have been the subject of greater regulatory scrutiny—particularly in regions where lovastatin is only available as a prescription. Some believe selling a product containing monacolin K is equivalent to selling a prescription pharmaceutical product. The US FDA determined in 1998 that RYR products that contain more than trace amounts of monacolin K are unapproved new drugs and cannot be sold legally as dietary supplements [14]. The European Food Safety Authority (EFSA) does not permit the sale or supply of RYR preparations that contain ≥3 mg of monacolin K [153]. The 2021 European Society of Cardiology guidelines on cardiovascular disease prevention in clinical practice recommend against using RYR extract [154].

In summary, RYR preparations have been shown to be safe and effective in improving lipid profiles and, to some extent, in reducing the risk of cardiovascular events. However, they should not replace conventional treatments (statins, ezetimibe, and PCSK9 inhibitors), as higher-quality long-term outcomes data exist. Of note, RYR extract may be considered in: (1) patients with statin intolerance, (2) patients with dyslipidemia ineligible for statin therapy, and (3) patients with a strong preference for RYR extract over conventional treatment. When recommending RYR products to patients, it is important to ensure that the product has been produced according to the principles of good manufacturing practice, to ensure consistency of dose of the active ingredient and the absence of harmful contaminants [14].

### 3.2. Berberine

Berberine is a quaternary benzylisoquinoline alkaloid used in China [16,25]. It can be isolated from the bark, fruit, root, rhizome, and stem of various plant genera, including *Berberis*, *Coptis*, and *Hydrastis* [16]. Berberine inhibits PCSK9, resulting in less degradation of the hepatic LDL receptor and improved clearance of serum LDL-C [23]. Berberine also upregulates the hepatic LDL receptor and thus serum LDL-C clearance at the level of the messenger ribonucleic acid by activating the Jun amino-terminal kinase and extracellular signal regulated kinases [16,24]. Other secondary mechanisms involve decreased gastrointestinal reabsorption of cholesterol, enhanced fecal excretion, and increased hepatic bile acid formation [25]. Berberine activates adenosine monophosphate-activated protein kinase (AMPK), which inhibits cholesterol and triglyceride synthesis, promotes hepatic fatty acid oxidation, and reduces lipogenic gene expression [26,27]. Berberine also inhibits nicotinamide adenine dinucleotide phosphate, which is involved in cholesterol synthesis [28]. The typical dosage of berberine is 200 to 1500 mg per day [29].

The expected LDL-C reduction is about −15 to −20% [16]. A meta-analysis of 19 RCTs and cross-sectional trials with 1372 participants found that berberine reduced TC (−1.17 mmol/L; 95% CI, −1.28 to −1.06), LDL-C (−1.06 mmol/L; 95% CI, −1.17 to −0.96) and TG (−0.60 mmol/L; 95% CI, −0.71 to −0.50) and increased HDL-C (0.24 mmol/L; 95% CI, 0.14–0.34) [29].

Side effects include gastrointestinal symptoms, headaches, and elevated transaminases [30,31]. No serious liver injury was reported in adults [16,29,31]. Kernicterus may be more common in infants taking berberine [31]. Pregnant women, breastfeeding mothers, and infants should avoid berberine. Interactions are possible with anticoagulants, anti-platelets, antidiabetic drugs, antihypertensive drugs, central nervous system depressants, cyclosporine, CYP substrates (CYP2C9, CYP2D6, and CYP3A4), dextromethorphan, and tacrolimus [22].

### 3.3. Bergamot (Citrus bergamia)

Bergamot (*Citrus bergamia Risso*) is a Mediterranean citrus fruit that is rich in flavonoids, which can be purified from the peel to produce bergamot polyphenolic fraction (BPF) [16,33]. These flavonoids can inhibit HMG-CoA reductase and acyl-coenzyme A cholesterol acyltransferase, which are involved in cholesterol production and transportation, respectively [16]. BPF inhibits pancreatic cholesterol ester hydrolase, which is involved in the esterification of cholesterol [32]. Other flavonoids, such as melitidin, neoeriocitrin, and rutin, have been shown to inhibit LDL-C oxidation and to inhibit cholesterol triglyceride synthesis via AMPK [26]. These flavonoids may potentially have anti-atherosclerotic properties through radical-scavenging activity [16]. BPF may also reduce intestinal absorption of cholesterol via increased fecal excretion of bile acids [16,33]. The typical dosage is 500 to 1500 mg per day [16].

The expected LDL-C reduction is about −7 to −40%. In a systematic review of 12 studies with 870 participants, BPF decreased TC (−12.3 to −31.3%), LDL-C (−7.6 to −40.8%), and TG (−11.5 to −39.5%) and increased HDL-C (1.0 to 6.5%). The authors found a dose-dependent response, with higher doses of BPF associated with increased lipid-lowering reductions [34].

Bergamot is associated with gastrointestinal discomfort and muscle cramps. Safety data on patients who are pregnant or breastfeeding are scant. There may be interactions with antidiabetic drugs due to hypoglycemia [22].

### 3.4. Garlic Extract

Garlic is an herb used in traditional Indian and Chinese medicine and is sold as raw garlic, extracted oil, or powdered tablets. It contains allicin (diallyl thiosulfinate), which inhibits HMG-CoA reductase, acetyl-CoA synthetase, squalene-monooxygenase, and potentially non-acetylated CoA [66]. Garlic may inhibit intestinal absorption of fatty acids and cholesterol and increase the excretion of bile acids [16]. The typical dosage ranges from about 0.3 to 20 g [16,67].

The expected LDL-C reduction is −5 to −10% [16]. In a recent network meta-analysis of 26 RCTs with 1620 participants, those in the garlic arm showed a decrease in TC (−0.25 mmol/L; 95% CI, −0.38 to −0.11), LDL-C (−0.21 mmol/L; 95% CI, −0.31 to −0.11), and TG (−0.14 mmol/L; 95% CI, −0.22 to −0.05) and an increase in HDL-C (0.03 mmol/L; 95% CI, 0.00 to 0.07) compared to placebo [15]. In another meta-analysis of six trials, garlic extract was shown to increase serum Lp(a) (54.59%; 95% CI, 30.47 to 78.71) in the trials that lasted longer than 12 weeks [155].

Garlic extract has been shown to affect coronary plaque. In an RCT, 55 participants with metabolic syndrome were randomized to supplementation with aged garlic extract versus placebo and followed for a year. There was a relative reduction of about 30% in mean low-attenuation plaque percent (−1.5% ± 2.3; *p* < 0.01) on cardiac computed tomography angiography, when compared to baseline. However, there were no changes in the total plaque volume, dense calcium, or non-calcified plaque between the two groups [156]. Another recent RCT focused on aged garlic extract in participants with DM2. After 1 year follow up, those in the supplementation arm saw a similar 29% relative reduction in low-attenuation plaque percent compared to their baseline on cardiac computed tomography angiography. When compared to placebo, those in the supplementation arm also had a higher reduction in median low-attenuation plaque percent (−0.02% ± 18.8; *p* = 0.0415). However, there were no changes in the volumes of total plaque, fibrous plaque, or fibrofatty plaque between the two arms [157].

Garlic extract is associated with gastrointestinal symptoms, body odor, garlicy breath, aftertaste, and increased bleeding risk [16,68]. It is possibly unsafe when used in higher levels in patients who are pregnant or breastfeeding. Garlic extract may have potential interactions with anti-hypertensive drugs, anti-diabetic drugs, atazanavir, CYP2E1/CYP3A4 inducers and inhibitors, isoniazid, protease inhibitors, saquinavir, tacrolimus, and warfarin [22].

### 3.5. Artichoke Leaf Extract

Artichokes are native to the Mediterranean region and can be a part of the Mediterranean diet. Many antioxidants can be derived from artichoke leaf extract, including caffeic acid, flavonoids, volatile sesquiterpene, and mono- and dicaffeoylquinic acid (cynarin and chlorogenic acid) [16,21,158]. There are two proposed mechanisms of action. Flavonoids, such as luteolin, may inhibit HMG-CoA reductase, which is involved in cholesterol synthesis [16,20]. They may also induce pathways involving the sterol regulatory element-binding proteins (SREBP) and the acyl-coenzyme A cholesterol acyltransferase, resulting in a reduction in cholesterol [20]. There may also be an increase in the gastrointestinal excretion of bile acids. The typical dosage for artichoke leaf extract can range from 500 to 2800 mg per day [21].

The expected LDL-C reduction is about −5 to −15% [16]. A network meta-analysis of 11 RCTs of artichoke leaf extract with 775 participants found a reduction in TC (−0.46 mmol/L; 95% CI, −0.69 to −0.23), LDL-C (−0.39 mmol/L; 95% CI, −0.54 to −0.24), and TG (−0.19 mmol/L; 95% CI, −0.32 to −0.06). There were no significant changes with HDL-C [15].

Reported side effects include gastrointestinal discomfort, skin reactions, and potential asthma exacerbations [16,22]. However, there are no reports of significant side effects [21]. Data on use in pregnant or breastfeeding patients are insufficient. There may be interactions with antidiabetic drugs, antihypertensive drugs, and CYP2B6 and CPY2C19 inducers and inhibitors [22].

### 3.6. Green Tea

Green tea is derived from the leaves of the *Camellia sinensis* plant and can be consumed as tea or green tea extract. Green tea is rich in catechins, which are flavonoids and include epicatechin, epicatechin-3-gallate, epigallocatechin, epigallocatechin-3-gallate, gallocatechin, catechin gallate, and gallocatechin gallate [159]. There are multiple potential mechanisms of action for green tea. It inhibits the expression of inducible nitric oxide synthase [69]. Green tea also activates AMPK and inhibits HMG-CoA reductase [16,69]. It is postulated that green tea inhibits the reabsorption of bile acids in the ilium, resulting in the increased biliary excretion of cholesterol and higher LDL receptor expression on the liver [16,70]. The typical dosage ranges from 100 to 500 mg per day [16,69].

The average expected decrease in LDL-C is about −5% [16]. A network meta-analysis of 25 RCTs with 1487 participants comparing green tea extract versus placebo revealed that those in the green tea arm had a significant decrease in TC (−0.18 mmol/L; 95% CI, −0.33 to −0.04) and LDL-C (−0.17 mmol/L; 95% CI, −0.28 to −0.07). There were no significant changes in HDL-C or TG [15].

Green tea extract is generally well tolerated. Common side effects include gastrointestinal discomfort, transitory blood pressure elevations, and rashes [16,69]. Rare serious adverse effects include thrombotic thrombocytopenic purpura, hepatotoxicity, or hypokalemia [22]. At higher doses, green tea may lead to iron and folate deficiency by binding and reducing their absorption. In turn, special precautions may need to be taken in pregnant patients [16]. There may be an increased risk of miscarriage when consuming 200 mg or more of caffeine per day [71]. There may be some interactions with 5-fluorouracil, adenosine, anti-coagulants, anti-platelet drugs, antidiabetic drugs, statins, beta agonists, bortezomib, carbamazepine, cimetidine, clozapine, lisinopril, lithium, stimulants, verapamil, valproate acid, and more [22].

### 3.7. Olive Extract

Olive trees (*Olea europaea*) are native to the Mediterranean, and their fruits and leaves can be used to create olive extract [93,94]. The extract contains phenolic compounds, such as hydroxytyrosol, L-tyrosol, and secoiridoid oleuropein [93]. Olive extract reduces lipid peroxidation, increases bile excretion, and inhibits HMG-CoA reductase and acetyl-CoA cholesterol acyltransferase activity [92]. The typical dosage can range up to about 136.2 mg oleuropein and 6.4 mg hydroxytyrosol per day [93].

In a randomized, double-blind, controlled, crossover trial, 60 participants with hypertension were randomized into the placebo versus the olive leaf extract arm for 6 weeks. Participants then underwent a 4 week washout period and were assigned to the crossover arm for an additional 6 weeks. The authors found that olive leaf extract resulted in reductions of TC (−0.32 ± SD 0.70 mmol/L; *p* = 0.002), LDL-C (−0.19 ± SD 0.56 mmol/L; *p* = 0.017) and TG (−0.18 ± SD 0.48; *p* = 0.008) compared to the control arm. However, there were no significant changes in HDL-C [93].

There are no known adverse effects of olive extract. Although presumably safe to consume during pregnancy and while breastfeeding, there are no known data on the levels consumed through olive extract. There are no known interactions with medications [94].

### 3.8. Gamma-Oryzanol

Gamma-oryzanol is extracted from the bran of rice (*Oryza sativa* L.), which has been used in Islamic traditional medicine. The main mechanism of cholesterol reduction is via inhibition of gastrointestinal cholesterol absorption and increased fecal excretion of bile acids. Gamma-oryzanol has been shown to inhibit HMG-CoA reductase and platelet aggregation [95]. Gamma-oryzanol may also affect serum lipid levels by altering the gut microbiome [96]. The typical dosage ranges from 100 to 300 mg daily [16,22].

The expected LDL-C reduction is about −5 to −10% [16]. In a meta-analysis of 14 studies with 270 participants being treated with rice bran oil, those in the nutraceutical arm had a decrease in TG (−0.10 mmol/L; 95% CI, −0.20 to −0.004), TC (−0.19 mmol/L; 95% CI, −0.29 to −0.11), and LDL-C (−0.20 mmol/L; 95% CI, −0.29 to −0.04). There was no significant change in HDL-C [97].

There are no reported side effects with gamma-oryzanol [16,97]. There are no safety data on patients who are pregnant or breastfeeding, and there are no known drug interactions [22].

### 3.9. Soybean Protein

Soybean, also known as *Glycine max*, is a legume that traditionally has been consumed in Asian countries but has seen an increased adoption in the West. It contains high protein, polyunsaturated fatty acid (PUFA), and fiber [160]. Soy protein has many potential lipid-lowering mechanisms. β-conglycinin, a soy peptide, inhibits HMG-CoA reductase and reduces the amount of PCSK9 protein in the liver [141]. Soybean protein hydrolysate has been shown to reduce cholesterol absorption via increased bile acid excretion, cholesterol synthesis inhibition, and increased transcription of the LDL receptor [142,143]. Soy protein has been shown to increase apoB receptor activity and to decrease hepatic synthesis of cholesterol and lipoprotein secretion [143]. Soybeans have also been shown to alter the gut microbiome by increasing *Bifidobacteriaceae*, *Deferribacteraceae*, and *Clostridiales*, which may play a role in soy protein’s lipid-lowering properties [144]. The typical dosage ranges from 25 to 120 mg daily [16,69].

The expected LDL-C reduction is about −3 to −10% [16]. A meta-analysis of 43 clinical trials with 2607 total participants that were given either dietary or supplementary soy versus placebo found that those in the soy arm experienced a decrease in TC (−0.17 mmol/L; 95% CI, −0.24 to −0.09) and LDL-C (−0.12 mmol/L; 95% CI, −0.17 to −0.07). The authors did not find a dose-dependent correlation with the lipid-lowering effect [161]. Another meta-analysis of 10 RCTs with 973 participants found that soy isoflavone supplementation did not significantly change Lp(a) [162].

Common side effects include gastrointestinal discomfort, menstrual complaints, headaches, dizziness, and rashes [22]. Chronic use of higher doses of soy protein may affect fertility and thyroid function [16,69]. There may be decreased absorption of divalent and trivalent metals, such as calcium, copper, iron, magnesium, and zinc, due to phytic acid in soybeans [143]. Due to the potential estrogen effects, supplemental soy protein should be avoided during pregnancy. However, there are no limitations in dietary soy. Use of soy is likely safe during breastfeeding. There may be interactions with antidiabetic drugs, antihypertensive drugs, diuretics, estrogens, progesterone, tamoxifen, levothyroxine, monoamine oxidase inhibitors, and warfarin [22].

### 3.10. Lupin Protein

Lupin is another legume that grows natively in Australia, the Mediterranean region, and South America. Lupin protein inhibits both HMG-CoA receptor and PCSK9 activity [75,76]. It upregulates SREBP-2 via phosphatidylinositol-3-kinase, alpha serine/threonine-protein kinase, and glycogen synthase kinase-3 beta kinase pathways in the liver [77]. The typical dosage is about 35 g or less per day [78].

The expected LDL-C reduction is about –5 to −12% [16]. In an RCT where 33 patients with dyslipidemia were randomized to receive 25 g per day of lupin protein isolate versus placebo, LDL-C (−0.35 ± 0.54 mmol/L) decreased in patients with TC > 6.6 mmol/L [78].

The most common side effects include gastrointestinal discomfort. It is likely safe to use in patients who are pregnant or breastfeeding. There are no known interactions with drugs [22].

### 3.11. Policosanol

Policosanol is extracted from sugarcane (*Saccharum officinarum* L.) wax, wheat germ, beeswax, and rice. It is a mixture of aliphatic alcohols, including tetratriacontanol, dotriacontanol, triacontanol, tetracosanol, hexacosanol, heptacosanol, octacosanol, and nonacosanol [116]. Policosanol promotes bile acid production and lipolysis via inhibiting the expression of farnesoid X receptor-small heterodimer partner and activating the Takeda G-coupled protein receptor 5-AMPK signaling pathway, which inhibits HMG-CoA reductase activity [114,115]. The typical dose ranges from 5 to 20 mg [116].

In a meta-analysis of 22 RCTs with 1886 participants with dyslipidemia, policosanol supplementation was associated with a decrease in TC (−0.58 mmol/L; 95% CI, −0.87 to −0.30) and LDL-C (−0.71 mmol/L; 95% CI, −1.02 to −0.40) and an increase in HDL-C (0.13 mmol/L; 95% CI, 0.09 to 0.16). There was no significant change in TG [116].

Common side effects of policosanols include gastrointestinal discomfort, tachycardia, myalgia, hypertension, headache, dizziness, somnolence, insomnia, polydipsia, nocturia, dry skin, rashes, and weight gain [116]. Animal models appear to show that policosanol is safe to use in pregnancy [117,118]. Data on the safety of policosanol in patients who are breastfeeding remain insufficient. There are possible interactions with antidiabetic drugs, beta-blockers, nitroprusside, and warfarin [22].

### 3.12. Phytosterols

Phytosterols are plant-derived compounds that are found in all plant foods, particularly in vegetable oils, nuts, seeds, and grains [163]. Phytosterol is a broad term that encompasses both plant sterols and stanols, and the most common phytosterols are campesterol, sitosterol, and stigmasterol [110,163]. Their structure is similar to that of cholesterol. Phytosterols inhibit cholesterol absorption in the gastrointestinal tract by competing with dietary cholesterol in the formation of dietary micelles, decreasing secretion of apoB from enterocytes and hepatocytes, and increasing cholesterol efflux via increased expression of adenosine triphosphate-binding cassette (ABC) protein A1 and ABCG1 levels [110,111,112]. The typical dosage ranges from 400 mg to 3 g per day [16,110].

The expected LDL-C reduction is about –8 to −16% [16,69]. In a meta-analysis of eight RCTs with 297 participants with dyslipidemia, plant sterol and stanol supplementation was found to lower LDL-C (−0.31 mmol/L; 95% CI, −0.39 to −0.23). The authors found that there were no significant differences between supplemented or dietary phytosterols [110]. In another larger meta-analysis of 51 clinical trials with 3786 participants who were randomized to dietary phytosterols versus placebo, there was a decrease in LDL-C (−0.34 mmol/L; 95% CI, −0.38 to −0.30) and TG (−0.05 mmol/L; 95% CI, −0.09 to −0.01) in the phytosterol arm. The increase in HDL-C was not significant [163].

Plant sterols/stanols had the highest certainty of evidence as assessed by the Journal of Clinical Epidemiology (JCE) Grading of Recommendations Assessment, Development and Evaluation (GRADE) [15]. This was also reflected in recent ILEP guidelines that gave IIa recommendations, which acknowledged the moderate effects and quality of trials [69].

Phytosterols are generally well tolerated and safe [16,69]. Phytosterols are safe to use in pregnancy [113]. However, data on their use in those breastfeeding are insufficient. There are no known drug interactions that are clinically significant [22].

### 3.13. Viscous Dietary Fibers

Viscous dietary fibers are an umbrella term used for a variety of complex polysaccharides found in oats, barley, legumes (lentils, lima beans, kidney beans, and pinto beans), fruits (apples, pears, plums, and citrus fruits), and vegetables (broccoli, Brussels sprouts, carrots, and green peas), seeds, and nuts that are resistant to digestion in the small intestine [164]. The US FDA and EFSA have both confirmed the lipid-lowering capabilities of soluble fiber [60,165]. Soluble fibers have different mechanisms of action and efficacy in their lipid lowering capacity [164].

#### 3.13.1. β-Glucan

β-glucan is a soluble fiber found in the endospermic cell walls of oats and is produced during the ripening process. Due to the increased viscosity of β-glucan, this reduces the absorption of cholesterol from the gastrointestinal tract. It also increases gastrointestinal elimination via bile acid excretion [59]. Typical daily consumption ranges from 3 to 25 g [16,60].

In a meta-analysis of 13 RCTs that randomized 927 patients with dyslipidemia to receive a diet enriched with oat β-glucan versus placebo, the authors found a decrease in TC (−0.24 mmol/L; 95% CI, −0.28 to −0.20) and LDL-C (−0.27 mmol/L; 95% CI, −0.35 to −0.20). However, there was no significant change in TG or HDL-C [60].

β-glucans are typically well tolerated. Data on their use in patients who are pregnant or breastfeeding are insufficient. There may be interactions with antihypertensives and immunosuppressant drugs [22].

#### 3.13.2. Psyllium

The husk of *Plantago* seed contains psyllium, which is a soluble fiber that forms a viscous gel. Psyllium reduces absorption of cholesterol via the gastrointestinal tract and binds to bile acids, which increases intestinal excretion of cholesterol and increases hepatic LDL receptor expression. Typical consumption can range from 2 to 20 g per day [63].

In a meta-analysis of 28 clinical trials involving 1924 patients with and without dyslipidemia, the authors found a reduction in LDL-C (−0.33 mmol/L; 95% CI, −0.38 to −0.27), non-HDL-C (−0.39 mmol/L; 95% CI, −0.50 to −0.27), and apoB (−0.05 g/L; 95% CI, −0.08 to −0.03) [63].

Side effects include gastrointestinal discomfort, mild anaphylactic allergic reactions, bowel obstructions, and esophageal obstructions [16,22,64]. Increased water consumption reduces obstructions [164]. Psyllium is safe to use during pregnancy or while breastfeeding [65]. There may be drug interactions with carbamazepine, digoxin, estradiol, lithium, metformin, and olanzapine [22].

#### 3.13.3. Glucomannan

Glucomannan is a dietary fiber found in the tuber root of *Amorphophallus konjac*, which has been used as both food and medicine in Asia. It inhibits HMG-CoA reductase and reduces absorption of cholesterol in the gastrointestinal tract [57]. Glucomannan also increases the conversion of bile acids into cholesterol through increased 7-α-hydroxylase activity [16]. The typical dosage ranges from 1 to 15 g per day [16,58].

In a meta-analysis of 12 RCT with 370 participants with and without dyslipidemia that were treated with dietary glucomannan versus placebo, the authors found a decrease in LDL-C (−0.35 mmol/L; 95% CI, −0.46 to −0.25) and non-HDL-C (−0.32 mmol/L; 95% CI, −0.46 to −0.19). However, there was no significant change in apoB [58].

Glucomannan is associated with gastrointestinal discomfort and obstructions in the bowel or esophagus [22,58]. It may also reduce the absorption of vitamin E. Data on its use in patients who are pregnant or breastfeeding are insufficient. There may be issues with the absorption of other medicines when glucomannan is used [22].

#### 3.13.4. Guar Gum

Guar, also known as *Cyamopsis tetragonoloba* or *Cyamopsis psoraloides*, is natively grown in India, Pakistan, North Africa, and South America [61,166]. It contains guar gum, which is a fermentable and soluble fiber consisting of galactose and mannose. Guar gum has a wide range of applications, as it is used in food (emulsifiers or thickeners), cosmetics, paper, textiles, toiletry, water treatment, and solar cells [166]. It works by preventing absorption of cholesterol in the gastrointestinal tract and increasing bile acid extraction. The typical dosage ranges from 30 to 100 g per day [61].

In a meta-analysis of 25 clinical trials with 1095 participants, there was a decrease in TC (−0.53 mmol/L; 95% CI, −0.69 to −0.36) and LDL-C (−0.45 mmol/L; 95% CI, −0.61 to −0.29). There was no significant change in TG or HDL-C [61].

Guar gum is generally well tolerated, and side effects include gastrointestinal discomfort and obstruction of the esophagus or bowel [22,61]. It is safe to use during pregnancy, as it treats intrahepatic cholestasis of pregnancy [62]. However, data on its use in patients who are breastfeeding remain insufficient. There may be interactions with penicillin, metformin, estradiol, and digoxin. It also may inhibit the absorption of other oral drugs [22].

#### 3.13.5. Pectin

Pectins are soluble fibers that are found in citrus and apples. They can be commercially extracted from the pulp waste in the juice pressing process. They are used as thickeners and food stabilizers in jams, marmalades, yogurts, desserts, and more [107]. Pectins work by decreasing cholesterol absorption in the gastrointestinal tract and increasing excretion of bile acids [107,108]. They also decrease HMG-CoA reductase and increase cholesterol 7-α-hydroxylase in the liver. Pectins also modulate the gut microbiome by increasing *Lactobacillus*, *Bifidobacterium*, and *Bacteroides* [108]. Typical consumption can be about 6 g per day [107].

Various degrees of esterification of pectins, molecular weight, and sourcing from apples or citrus can impact the lipid-lowering capacity of pectins [107]. The expected LDL-C reduction is about −5 to −10% [16,107]. In a randomized, crossover design trial of 90 participants with dyslipidemia, the authors found a decrease in TC (−5.41 ± 7.27%) and LDL-C (−8.05 ± 9.31%) in patients treated with pectins from citrus with high degrees of esterification. Similarly, there was a decrease in TC (−6.54 ± 6.49%) and LDL-C (−9.26 ± 9.71%) with pectins from apples with high degrees of esterification. The authors found that pectins with higher degrees of esterification lowered TC and LDL-C more effectively than pectins with lower degrees of esterification. They also found that there was no significant difference in the TC and LDL-C lowering capabilities of pectins from citrus or apples. There was no significant change in TG or HDL-C from any type of pectin [107].

Pectins are generally well tolerated. Gastrointestinal discomfort is common in citrus pectins with higher degrees of esterification compared to pectins from apples [107]. Precautions should be taken in patients who are pregnant, as pectins bind to vitamin B12. This may exacerbate vitamin B12 deficiencies and potentially lead to higher risks of spina bifida in fetuses [109]. Pectins should be safe to use in patients who are breastfeeding. There may be interactions with digoxin, lovastatin, and tetracyclines [22].

### 3.14. Nigella Sativa

*Nigella sativa* (*N. sativa*), also known as black caraway, black cumin, or black seed, is a flower that is grown natively in Eastern Europe, the Middle East, North Africa, and South Asia [90]. It has been used as Islamic traditional medicine and has many modern applications, as it is used in cosmetics, food, and pharmaceuticals. *N. sativa* contains thymoquinone, flavonoids, and PUFAs such as linoleic and oleic acids. These can be made into a powder, extract, or oil suspension [89,90]. *N. sativa* works by reducing gastrointestinal absorption of cholesterol, increasing biliary excretion, reducing cholesterol synthesis, inhibiting lipid oxidation, and upregulating LDL receptors [89]. Typical daily doses can range from 200 mg to 3 g for powders, capsules, and extracts or 1 to 5 mL for oil suspensions [90].

In a meta-analysis of 37 trials with 2531 participants, *N. sativa* supplementation was associated with a reduction in TC (−0.43 mmol/L; 95% CI, −0.54 to −0.32), LDL-C (−0.48 mmol/L; 95% CI, −0.58 to −0.37), and TG (−0.18 mmol/L; 95% CI, −0.23 to −0.12) and an increase in HDL-C (0.05 mmol/L; 95% CI, 0.03 to 0.07) [90].

*N. sativa* is well tolerated and generally safe. It may be associated with gastrointestinal discomfort and elevated alkaline-phosphate, aspartate-aminotransferase, alanine-aminotransferase, and gamma-glutamyl transferase [89]. Despite this, *N. sativa* appears to be safe to use during pregnancy [91]. The data on its use during breastfeeding remain insufficient. There may be possible drug interactions with anticoagulants, anti-platelet drugs, antidiabetic drugs, antihypertensives, cyclosporine, diuretics, immunosuppressants, and serotonergic drugs [22].

### 3.15. Silymarin

Silymarin contains a group of flavonolignans, including silibinin, silybin, silydianin, silychristin, and isosilybin [137]. It is extracted from the fruit of *Silybum marianum*, also known as milk thistle, which is native to Europe [138]. Silymarin has been shown to increase lipolysis and β-oxidation via the upregulation of carnitine palmitoyl transferase 1 (CPT1) [135]. It has also been shown to increase cholesterol efflux via the increased expression of ABCA1 [136]. The typical dosage ranges from 140 to 700 mg per day [137].

In a meta-analysis of 11 RCTs of 816 participants with diabetes or liver disease, silymarin supplementation was found to decrease TC (−0.45 mmol/L; 95% CI, −0.80 to −0.10), LDL-C (−0.27 mmol/L; 95% CI, −0.49 to −0.05), and TG (−0.29 mmol/L; 95% CI, −0.53 to −0.05) and to increase HDL-C (0.09 mmol/L; 95% CI, 0.02 to 0.15) [137].

Silymarin is typically well tolerated. However, side effects of silymarin include gastrointestinal discomfort, headache, ureteric calculi, and hemolytic anemia. Reported serious adverse events include transient ischemia attack, MI, and cardiovascular death [137]. Safety data on its use in patients who are pregnant or breastfeeding are not available [138]. There are possible interactions with antidiabetic drugs, morphine, tamoxifen, sirolimus, and warfarin [22].

### 3.16. Sea Buckthorn

Sea buckthorn (*Hippophae rhamnoides* L.) is a plant found natively in northeastern Europe, Russia, China, Tibet, and Mongolia. It has been used in traditional Tibetan medicine and has been gaining popularity as a fruit juice [133]. It contains flavonoids, including isorhamnetin, kaempferol, and quercetin [132,133]. They increase the expression of peroxisome proliferator-activated receptor (PPAR)-α, PPAR-γ, and ABCA1 and decrease the expression of SREBP-2. Sea buckthorn promotes the expression of CPT1A, which is involved in increasing lipolysis and β-oxidation [132]. The typical dose of sea buckthorn seed oil is about 0.75 mL [133].

In a meta-analysis of 13 RCTs with 1167 participants, sea buckthorn supplementation decreased TC (−0.35 mmol/L; 95% CI, −0.64 to −0.05), TG (−0.72 mmol/L; 95% CI, −1.13 to −0.32), and LDL-C (−0.40 mmol/L; 95% CI, −0.76 to −0.04) and increased HDL-C (0.37 mmol/L; 95% CI, 0.06 to 0.68) [133].

Sea buckthorn is generally well tolerated. Animal models appear to show that sea buckthorn is safe to use in pregnancy [134]. However, there are limited data on its use in patients who breastfeed. There may be drug interactions with anticoagulants, anti-platelets, and antihypertensives [22].

### 3.17. Anthocyanins

Anthocyanins are antioxidants and are red, blue, or purple pigmented flavonoids [18]. They are used in food coloring and are found in fruits, flowers, roots, stems, leaves, and vegetables of blueberries, black rice, cherries, purple cabbage, purple grapes, and raspberries [16,18]. They have been shown to downregulate the messenger ribonucleic acid expression of SREBP-1c and fatty acid synthase, resulting in less fat accumulation in adipocytes [17]. Typical dosages range from 100 to 450 mg daily [18].

The expected LDL-C reduction is about −5 to −10% [16]. In a meta-analysis of 20 trials with 1311 participants, the authors found a decrease in LDL-C (−0.19 mmol/L; 95% CI, −0.31 to −0.06) and TG (−0.20 mmol/L; 95% CI, −0.33 to −0.07) and an increase in HDL-C (0.09 mmol/L; 95% CI, 0.02 to 0.15). There was no significant change in TC [18].

Anthocyanins are generally well tolerated [16]. However, precautions should be taken with anthocyanins during pregnancy. There was a single case report of polyhydramnios discovered at 37 weeks of gestation from the prenatal closure of the ductus arteriosus after the maternal ingestion of MonaVie, which is a juice blend containing anthocyanins [19].

### 3.18. Spirulina

*Arthrospira maxima*, known commercially as spirulina, is a cyanobacterium containing amino acids, beta-carotene, polyphenols, vitamins, and PUFA [140]. C-phycocyanin, the main component involved in the lipid-lowering effect of spirulina, is a photosynthetic pigment that is used as a natural blue food coloring. The mechanism of action involves activating heme oxygenase-1, which is an atheroprotective enzyme involved in the heme catabolic pathway in endothelial cells [16,69]. C-phycocyanin also has antioxidant, anti-inflammatory, and radical-scavenging properties [16]. PUFAs from spirulina affect the gut microbiome, which may alter lipid metabolism [139]. The typical dosage ranges from 1 to 10 g per day [69].

The average expected decrease in LDL-C is about −5 to −15% [16,69]. One meta-analysis of eight clinical trials with 420 patients with diabetes found a decrease in TC (−0.30 mmol/L; 95% CI, −0.55 to −0.05), TG (−0.19 mmol/L; 95% CI, −0.34 to −0.04), and LDL-C (−0.24 mmol/L; 95% CI, −0.41 to −0.07). However, there was no significant change in HLD-C [15].

Side effects include gastrointestinal discomfort, bleeding, rashes, elevated transaminases, and cholestasis [22,140]. There are limited data on the safety in patients who are pregnant or breastfeeding. There may be interactions with anticoagulants, anti-platelet drugs, antidiabetic drugs, or immunosuppressant drugs [22].

### 3.19. Probiotics

Probiotics are live bacteria and yeast that are typically found in fermented foods and may alter the gut microbiome [122,123]. The most common microorganisms found in probiotics include *Lactobacillus*, *Bifidobacterium*, *Saccharomyces*, *Streptococcus*, *Enterococcus*, *Escherichia*, and *Bacillus* [123]. *Lactobacillus* and *Bifidobacterium* have been shown to increase the excretion of bile acids from the gastrointestinal tract via bile salt hydrolase enzymatic activity. This makes bile acids less water-soluble and thus more easily excreted by removing the amino acid moiety through deconjugation [119]. *Lacticaseibacillus* has also been shown to decrease Niemann–Pick C1-like 1 (NPC1L1) cholesterol transporter expression and increase cholesterol efflux via increased expression of ABCA1 [120]. *Lactobacillus rhamnosus* JL1 has also been shown to activate the AMPK pathway and inhibit PPAR-γ and SREBP-1C gene expression [121]. Probiotics can be delivered via capsules, yogurt, or fermented milk [122]. The typical dosage in capsules can range from 1 to 6 g per day [16,122].

The expected LDL-C reduction by probiotics is about −5% [16]. In a meta-analysis of 16 RCTs with 1429 non-obese participants with dyslipidemia, probiotics were associated with a decrease in TC (−0.34 mmol/L; 95% CI, −0.45 to −0.23) and LDL-C (−0.26 mmol/L; 95% CI, −0.36 to −0.17). No significant change was observed in TG or HDL-C [122].

Probiotics are generally safe and well tolerated. Side effects include gastrointestinal discomfort and infection, especially with probiotic formulations containing the yeast *Saccharomyces cerevisiae*. Probiotics are safe to use in infants and patients who are pregnant or breastfeeding [123].

### 3.20. Alpha Lipoic Acid

Alpha lipoic acid (ALA), also known as thioctic acid, is an antioxidant found in spinach, broccoli, tomatoes, red meat, and liver [72]. It is an essential cofactor for mitochondrial pyruvate dehydrogenase and α-ketoglutarate dehydrogenase [167]. ALA has been shown to modulate fat synthesis, mitochondrial β-oxidation of fat, clearance of TG-rich lipoproteins in the liver, and adipose TG accumulation [72]. The typical dosage ranges from 300 to 1800 mg per day [73].

In a meta-analysis of 12 RCTs with 548 patients, those in the ALA arm saw a decrease in TC (−0.28 mmol/L; 95% CI, −0.54 to −0.02), LDL-C (−0.28 mmol/L; 95% CI, −0.50 to −0.06), and TG (−0.35 mmol/L; 95% CI, −0.56 to −0.14). No significant changes in HDL-C were observed [73].

Adverse effects of ALA include gastrointestinal discomfort, skin rash, and rarely, insulin autoimmune syndrome. ALA is safe to use in patients who are pregnant [74]. However, there are insufficient data on its use in patients who are breastfeeding. ALA may interact with alkylating agents, anticoagulants, anti-platelet drugs, antidiabetic drugs, antitumor antibiotics, and levothyroxine [22].

### 3.21. Conjugated Linoleic Acid

Conjugated linoleic acid is a trans-fatty acid produced by bacteria in the gastrointestinal tract of ruminant animals via the metabolism of PUFAs and monounsaturated fatty acids. It is found in dairy products and meats from cows and sheep [49]. Conjugated linoleic acid promotes cholesterol efflux by increasing expression of cholesterol transporters ABCA1 and ABCG1 [48]. Typical dosages range from 0.5 to 7 g per day [49].

The expected LDL-C reduction is about −5% [16]. In a meta-analysis of 15 clinical trials, conjugated linoleic acid supplementation was associated with a decrease in LDL-C (−0.22 mmol/L; 95% CI, −0.36 to −0.08). There were no significant changes in TC, TG, or HDL-C [49].

Conjugated linoleic acid is associated with gastrointestinal discomfort and hepatotoxicity [50]. It is safe to use in patients who are pregnant [51]. However, there are insufficient data on its use in patients who are breastfeeding. Possible drug interactions include those with anticoagulants, anti-platelet drugs, and antihypertensives [22].

### 3.22. Chitosan

Chitosan is a polysaccharide derived from chitin, which is found in crustaceans [42,43]. Due to its positively charged amino group, chitosan interferes with gastrointestinal absorption by binding to negatively charged fatty acids and bile acids and disrupting the emulsification of neutrally charged cholesterol [42]. Typical dosages range from 0.3 to 3 g per day [43].

The expected LDL-C reduction is about –5% [16]. In a meta-analysis of 11 RCTs with 1011 participants, chitosan supplementation was associated with a decrease in TC (−1.39 mmol/L; 95% CI, −2.17 to −0.62), LDL-C (−0.83 mmol/L; 95% CI, −1.64 to −0.01), and TG (−1.06 mmol/L; 95% CI, −1.67 to −0.45). There was no significant change in HDL-C [43].

Chitosan should not be used in patients with allergies to crustaceans or shellfish [42]. Other side effects include gastrointestinal discomfort. There are insufficient data on its use in patients who are pregnant or breastfeeding. Possible drug interactions include those with acyclovir or warfarin [22].

### 3.23. Pantethine

Pantethine is a compound produced endogenously via pantothenic acid, which is also known as vitamin B5 [16,98]. The mechanism of action is believed to include cysteamine and involves the inhibition of HMG-CoA reductase and acetyl-CoA carboxylase, which are involved in TG synthesis and lipoprotein metabolism. However, further research is needed to elucidate the full mechanism. The typical dosage is about 600 to 1200 mg per day [98].

In a triple-blinded RCT with 30 participants, those in the pantethine arm saw a decrease in TC (−6%), LDL-C (−11%), and apoB (−8%). However, there were no significant changes in HDL-C, TG, or Lp(a). The main side effect of pantethine is gastrointestinal symptom [98]. Pantethine can be used in children and in patients with chronic kidney disease, including those on dialysis [99,100]. There are insufficient data on its use in patients who are pregnant or breastfeeding. There are no known drug interactions [22].

## 4. Nutraceuticals and Their Effects on Other Lipid Targets

### 4.1. Polyunsaturated n-3 Fatty Acids

Eicosapentaenoic acid (EPA) and docosahexaenoic acid (DHA) are long-chain PUFAs that are found naturally in fish, krill, squid, eggs, algae, flaxseeds, walnuts, clary sage, and various other edible seeds. The EFSA acknowledges that consumption of EPA and DHA may lower serum triglyceride levels [102]. These PUFAs work by reducing the synthesis of hepatic VLDL, endogenous fatty acids, substrates available for TG synthesis, and the activity of diacylglycerol acyltransferase or phosphatidic acid phosphohydrolase, which are involved in TG synthesis. They also promote β-oxidation of fatty acids and increase phospholipid synthesis [101].

Formulations can include over-the-counter supplements, which can contain higher levels of saturated fat. Prescriptions are purified pharmaceutical-grade formulations that contain less saturated fats. Both supplements and prescription formulations can contain a combination of EPA and DHA. However, only prescription formulations contain solely EPA. It should be noted that the over-the-counter supplements are not equivalent to the prescription formulations, as only purified EPA has also been shown to reduce plaque volume [168]. For both supplements and prescription formulations, the typical dosage is ≤4 g per day of EPA and DHA [102,103]. For prescription formulations containing only EPA, the typical dosage is ≤4 g per day [105].

In a meta-analysis of 46 RCTs with 4991 participants with DM2, those in the fish oil supplementation arm saw a decrease in TC (−0.22 mmol/L; 95% CI, −0.32 to −0.11) and TG (−0.36 mmol/L; 95% CI, −0.48 to −0.25) and an increase in HDL-C (0.05 mmol/L; 95% CI, 0.02 to 0.08). There was no significant change in LDL-C [169]. Another meta-analysis of 33 RCTs with 2704 participants with metabolic syndrome investigated the individual effects of DHA and EPA supplementation versus control. Those in the EPA supplementation arm saw a decrease in TC (−0.24 mmol/L; 95% CI, −0.43 to −0.05) and LDL-C (−0.13 mmol/L; 95% CI, −0.25 to −0.01). There was no significant change in HDL-C with EPA supplementation. In the DHA supplementation arm, there was a decrease in TG (−0.29 mmol/L; 95% CI, −0.37 to −0.21) and an increase in TC (0.14 mmol/L; 95% CI, 0.03 to 0.25), LDL-C (0.26 mmol/L; 95% CI, 0.15 to 0.38) and HDL-C (0.07 mmol/L; 95% CI, 0.04 to 0.09) [104].

There are strong data supporting the use of prescription EPA formulations. The Japan EPA Lipid Intervention Study (JELIS) trial was a large open-label RCT with 18,645 participants of Japanese descent with dyslipidemia. All patients were on 10 mg of pravastatin or 5 mg of simvastatin. They were randomized to receive 1.8 g per day of prescription EPA or placebo. The authors found a significant reduction in TG (23%) and major adverse cardiovascular events (MACE) (HR, 0.81; 95% CI, 0.69 to 0.95) in the EPA arm [170].

Another landmark RCT, the Reduction of Cardiovascular Events with Icosapent Ethyl–Intervention Trial (REDUCE-IT), further cemented the use of prescription EPA formulations in treating elevated triglycerides. A total of 8179 participants who had fasting TG between 1.53 to 5.64 mmol/L (135 to 499 mg/dL) and were already on a statin were randomized to 4 g of prescription EPA. The authors found a reduction in TG (−18.3%), HDL-C (−2.6%), non-HDL-C (−3.6%), high sensitive-CRP (−13.9), and MACE (HR, 0.75; 95% CI, 0.68 to 0.83) in the EPA arm [105].

Similar results have not been reproduced with omega-3 fatty acids containing both EPA and DHA. The Long-Term Outcomes Study to Assess Statin Residual Risk with Epanova in High Cardiovascular Risk Patients with Hypertriglyceridemia (STRENGTH) trial was a large RCT with 13,078 patients who were at high risk of cardiovascular disease. They were randomized to 4 g of omega-3 FA, consisting of both prescription EPA and DHA versus corn oil. This trial was terminated early due to futility as no difference was observed in MACE (HR, 0.99; 95% CI, 0.90 to 1.09; *p*  = 0.84) despite a significant decrease in TG (−19.0%; 95% CI, −39.2 to −6.4%) [103].

Prescription and supplemental DHA and EPA are associated with gastrointestinal discomfort and new-onset atrial fibrillation and atrial flutter. Those sourced from fish or krill can have a fishy aftertaste [103,105]. DHA and EPA are safe to use in pregnancy, as they are frequently in prenatal vitamins [106]. They are also likely safe to use during breastfeeding. Possible drug interactions include those with anticoagulants, anti-platelet drugs, antihypertensives, contraceptives, cyclosporine, and tacrolimus [22].

### 4.2. Niacin

Many clinicians use nicotinic acid (niacin or vitamin B_3_) in patients who are statin intolerant in the management of hypertriglyceridemia, despite it no longer being recommended for LDL-C reduction [1,7]. It inhibits diacylglycerol acyltransferase-2, which decreases TG synthesis and LDL-C by increasing hepatic apoB degradation. It raises HDL-C by stimulating hepatic apolipoprotein A-I production [84]. Typical dosages are ≤2 g daily [85,86].

The Atherothrombosis Intervention in Metabolic Syndrome with Low HDL/High Triglycerides: Impact on Global Health Outcomes (AIM-HIGH) trial was a large multi-center RCT that was terminated early due to the lack of efficacy. A total of 3414 participants with cardiovascular disease on simvastatin (and ezetimibe if LDL-C was >2.07 mmol/L [80 mg/dL]) were randomized to receive 1500 to 2000 mg niacin versus placebo. After 3 years, there was no difference in MACE (HR, 1.02; 95% CI, 0.87 to 1.21; *p* = 0.79) despite the niacin group experiencing an increase in HDL-C (25.0%) and a decrease in TG (−28.6%) and LDL-C (−12.0%) [86].

In the Heart Protection Study 2–Treatment of HDL to Reduce the Incidence of Vascular Events (HPS2-THRIVE) trial, 25,673 participants with established atherosclerotic cardiovascular disease were randomized to 2 g extended release niacin and laropiprant (used to minimize flushing from niacin) versus placebo. All participants received simvastatin (and ezetimibe if TC was ≥135 mg/dL or 3.5 mmol/L). Despite an increase in HDL-C (0.16 mmol/L) and a reduction in LDL-C (−0.25 mmol/L) and TG (−0.37 mmol/L) in the niacin arm, there were no significant differences in composite major vascular events (HR, 0.96; 95% CI, 0.90 to 1.03; *p* = 0.29). The authors did find a 10% reduction in revascularization procedures (RR, 0.90; 95% CI, 0.82 to 0.99; *p* = 0.03) in the niacin arm [85].

A meta-analysis of 17 studies with 35,760 participants with cardiovascular disease or dyslipidemia found that niacin monotherapy reduced the risk of acute coronary syndrome (RR, 0.74; 95% CI, 0.58 to 0.96), cerebral vascular accident (RR, 0.74; 95% CI, 0.59 to 0.94), and revascularization (RR, 0.79; 95% CI, 0.64 to 0.98) compared to participants not on statins [171]. Another meta-analysis of 14 RCTs with 9013 participants found that those in the niacin arm experienced a reduction in Lp(a) (−22.9%, 95% CI, −27.3 to −18.5) [172].

Due to the AIM-HIGH and HPS2-THRIVE trials, the US FDA withdrew the indication for extended-release niacin for co-administration with statins [173]. The European Medicines Agency has suspected all use of extended-release niacin and laropiprant, and niacin is no longer recommended to treat dyslipidemia in the European Union [7,174]. Similarly, the American multisociety guidelines recommend against using niacin for its LDL-C lowering capacity, but it may be considered in patients with severe hypertriglyceridemia [1].

Niacin is associated with increased gastrointestinal hemorrhage, peptic ulcers, myopathy, rhabdomyolysis, gout, flushing, skin lesions, skin infections, and lower respiratory infections. There is also an increased incidence of diabetes and hospitalizations for diabetes complications [85]. There are no restrictions with niacin for pregnant or breast feeding patients, as it is associated with a lower risk of congenital abnormalities [87]. There may be interactions with statins, isoniazid, and pyrazinamide [1,88].

### 4.3. L-Carnitine

L-carnitine is a hydrophilic quaternary ammonium cation that is found in meat, fish, poultry, and dairy. It is synthesized in the brain, liver, and kidneys [35]. L-carnitine supplementation, when concomitantly given with coenzyme Q10, may reduce SAMS [175]. L-carnitine decreases TG synthesis by decreasing available free fatty acids, increases mitochondrial oxidation of long-chain fatty acids, and increases production of apolipoprotein A1. The typical oral dosage ranges from 500 mg to 3 g and can be as high as 6 g per day [35].

In a meta-analysis of 55 RCTs with 3058 participants, those in the L-carnitine supplementation arm saw a reduction in TC (−0.22 mmol/L; 95% CI, −0.35 to −0.09), LDL-C (−0.14 mmol/L; 95% CI, −0.22 to −0.06), and TG (−0.11 mmol/L; 95% CI, −0.18 to −0.03) and an increase in HDL-C (0.04 mmol/L; 95% CI, 0.01 to 0.07). The authors found that higher doses of L-carnitine (≥2 g) yielded a greater reduction in TC and LDL-C [35]. Another meta-analysis of four RCTs with 218 participants found that oral L-arginine supplementation lowered Lp(a) (−0.09 mmol/L; 95% CI, −0.10 to −0.08) [176].

L-carnitine is generally well-tolerated. Patients may have fishy body odor, minor nausea, or other gastrointestinal discomfort [22,36]. L-carnitine supplementation in those who are pregnant and breastfeeding is safe, and parenteral carnitine supplementation is safe in infants [22,37,38]. There may be drug interactions with thyroid hormones and warfarin [22].

### 4.4. Vitamin E

Vitamin E is a fat-soluble vitamin with antioxidant properties. It has been shown to slow atherosclerotic plaque progression in patients with dyslipidemia when supplemented with vitamin C [177]. There are eight distinct chemical compounds, including α-, β-, γ-, and δ-tocopherol and α-, β-, γ-, and δ-tocotrienol [16]. Vitamin E lowers lipids by inhibiting HMG-CoA reductase, promoting scavenging for free radicals, and activating the PPAR -α, -β, and -γ receptors [145,146,147]. The typical dosage ranges from 400 to 800 UI per day [16].

In a meta-analysis of 16 RCTs with 803 participants, the authors found that vitamin E increased HDL-C (0.15 mmol/L; 95% CI, 0.07 to 0.23) compared to placebo. They found a dose-dependent response, with higher doses of vitamin E supplementation correlating with higher increases in HDL-C. There were no significant changes in TC, LDL-C, or TG [148].

Vitamin E supplementation is associated with an increased risk of bleeding, heart failure, hemorrhagic cerebral vascular accidents, prostate cancer, and all-cause mortality [147,149]. Vitamin E supplementation is safe in pregnant women, as it is frequently used in prenatal vitamins [106]. It is also likely safe during breastfeeding. There are possible drug interactions with alkylating agents, anticoagulants, anti-platelet drugs, cyclosporine, CYP3A4 substrates, niacin, and selumetinib [22].

## 5. Inconsistent Data Regarding Nutraceuticals and Dyslipidemia

### 5.1. Resveratrol

Resveratrol (3,5,40-trihydroxystilbene) is a non-flavonoid polyphenol compound primarily found in red grapes, raspberries, mulberries, blueberries, knotweed, and peanuts. It is also found in some juices and wines made from these fruits [129,130]. It potentially has antioxidant, anti-inflammatory, anti-apoptotic, and anti-cancer properties [130]. Resveratrol is an activator of the silent information regulation 2 homolog 1 and has been shown to suppress the hepatic upregulation of genes associated with lipogenesis and prevent the downregulation of genes involved in lipolysis [127]. It also may inhibit atherosclerosis by suppressing foam cell formation [128]. Typical dosages range from 250 to 3000 mg per day [129,130].

The LDL-C lowering effects of resveratrol have been mixed. One meta-analysis performed by Akbari et al. with 31 RCTs and 1722 participants investigated resveratrol in patients with metabolic syndrome and other related risk factors. Akbari et al. found a significant reduction in TC (−0.20 mmol/L; 95% CI, −0.33 to −0.06) but not in LDL-C, TG, or HDL-C. Notably, this meta-analysis included other pill combinations that contained other nutraceuticals in addition to resveratrol [178]. In another recent meta-analysis, Cao et al. only included RCTs where resveratrol was the sole nutraceutical used. Studies including other nutraceutical combinations with resveratrol were excluded. A total of 17 RCTs and 968 participants with metabolic syndrome and similar risk factors were included in their analysis. Cao et al. found in the resveratrol arm a significant reduction in TC (−0.27 mmol/L; 95% CI, −0.33 to −0.17), TG (−0.10 mmol/L; 95% CI, −0.14 to −0.05), and LDL-C (−0.147 mmol/L; 95% CI, −0.286 to −0.008) but not HDL-C. They also found a dose-dependent response with a higher reduction in LDL-C in those taking resveratrol for 12 weeks or longer and in those with diabetes [131].

Resveratrol may increase bleeding. It is likely safe to consume during pregnancy and breastfeeding, provided that the resveratrol is not consumed from beverages with alcohol. In patients with malignancies that grow in response to estrogen, such as breast, uterine, and ovarian cancer, it is advised that patients limit their intake due to resveratrol potentially acting on estrogen receptors. Resveratrol may have interactions with garlic, ginger, ginkgo, nattokinase, anticoagulants, anti-platelet drugs, and those involving the cytochrome P450 system, such as CYP1A1, CYP1A2, CYP1B1, CYP2C19, CYP2E1, and CYP3A4 [129].

### 5.2. Curcumin

Curcumin is a yellow polyphenolic compound found in the dried rhizomes of turmeric (*Curcuma longa*), which is natively grown in Southeast Asia [55,56]. It has been used in traditional Chinese and Indian medicine and has commercial applications, such as being used as a food additive or in cosmetics [179]. Curcumin inhibits intestinal NPC1L1 cholesterol transporter expression by inhibiting the SREBP2 transcription factor [52]. It also increases LDL-C clearance by increasing LDL receptor expression through the downregulation of PCSK9 expression [53]. Lastly, curcumin increases cholesterol efflux by upregulating ABCA1 expression [54]. Typical dosages can range from 50 mg to 6 g per day [55].

The expected LDL-C reduction is about −5% [16]. In a meta-analysis by Sahebkar et al. of five RCTs and 133 participants from a heterogeneous population, curcumin supplementation did not significantly change TC, TG, LDL-C or HDL-C [55]. However, another meta-analysis, by Altobelli et al., focused on patients with diabetes and included five RCTs with 476 participants. In the curcumin arm, there was a decrease in TC (−0.30 mmol/L; 95% CI, −0.53 to −0.07), LDL-C (−0.28 mmol/L; 95% CI, −0.52 to −0.04), and TG (−0.57 mmol/L; 95% CI, −0.83 to −0.31). However, there was no significant change in HDL-C [180]. Another meta-analysis, which included 10 RCTs with 683 participants, focused on patients with nonalcoholic fatty liver disease. Here, Khalili et al. found a decrease in TC (−0.81 mmol/L; 95% CI, −1.34 to −0.27) and TG (−0.49 mmol/L; 95% CI, −0.71 to −0.27) in the curcumin arm compared to placebo. However, no significant changes in LDL-C or HDL-C were found [179].

Curcumin supplementation is generally well tolerated and safe, and the most common side effect is gastrointestinal discomfort [22,56]. However, precautions should be taken in patients who are pregnant or breastfeeding, as levels greater than dietary levels may be unsafe. There may be drug interactions with alkylating agents, amlodipine, anticoagulants, anti-platelet drugs, antidiabetic drugs, CYP3A4 substrates, sulfasalazine, tacrolimus, talinolol, tamoxifen, and warfarin [22].

### 5.3. Magnesium

Magnesium is a naturally occurring mineral. Intravenous magnesium has been linked to lower left ventricular failure, lower mortality from ischemic heart disease, and lower all-cause mortality [82]. Magnesium downregulates HMG-CoA reductase expression and upregulates cholesterol 7α-hydroxylase and lecithin cholesterol acyltransferase expression [79]. Typical oral dosages can range from 35 to 500 mg per day [80].

In a meta-analysis of 12 RCTs with 677 participants with diabetes, magnesium supplementation lowered LDL-C (−0.18 mmol/L; 95% CI, −0.30 to −0.05) but did not significantly affect TC, HDL-C, or TG [80]. Other studies have not found a significant change in the lipid profile of patients without diabetes [81].

Side effects of hypermagnesemia include gastrointestinal discomfort, flushing, confusion, hypotension, hyperreflexia, respiratory depression, hyperkalemia, hypocalcemia, pulmonary edema, and cardiac arrest [82,83]. Magnesium is safe to use during pregnancy and actually has therapeutic indications when given at appropriate doses [22,83]. It appears safe when the total oral intake does not exceed 350 mg per day. There may be an increase in fetal mortality when intravenously used for tocolysis. There were no increases in fetal mortality when given for preeclampsia or eclampsia. When given intravenously or intramuscularly for more than 5 to 7 days, there may be an increased risk for neonatal fractures or osteopenia. Magnesium appears safe to use during breastfeeding. There may be drug interactions with aminoglycosides, antacids, bisphosphonates, calcium channel blockers, digoxin, ketamine, levodopa, carbidopa, potassium-sparing diuretics, quinolones, sulfonylurea, and tetracyclines [22].

### 5.4. Chromium

Chromium(III) is an essential trace mineral found in fruits, vegetables, grains, meat, beer, and wine [41]. It is involved in lipid metabolism, and chromium deficiency is associated with dyslipidemia [181]. Chromium upregulates gene expression of PPAR-γ and LDL receptor. Typical dosages range from 40 to 1000 mcg per day [39].

In a meta-analysis with 40 RCTs and 1966 participants, chromium supplementation was associated with only a decrease in TC (−0.17 mmol/L; 95% CI, −0.27 to −0.07). There were no significant changes in LDL-C, TG, or HDL-C [39]. In another meta-analysis with 24 RCTs focusing on 1418 patients with diabetes, chromium supplementation was found to decrease TC (−0.20 mmol/L; 95% CI, −0.29 to −0.11) and increase HDL-C (0.06 mmol/L; 95% CI, 0.01 to 0.11). There was no significant change in LDL-C or TG [40].

Chromium supplementation may lead to weight loss, hypoglycemia, anemia, thrombocytopenia, elevated transaminases, elevated creatinine, rhabdomyolysis, and dermatitis. Chromium supplementation is safe during pregnancy and in women breastfeeding. Drug interactions include those with levothyroxine, insulin, metformin, and other anti-diabetic medications [41].

### 5.5. Coenzyme Q10

Coenzyme Q10 (ubidecarenone, ubiquinone, or vitamin Q10) is a benzoquinone compound produced in the body. The highest concentrations are found in the heart, liver, kidneys, and pancreas [47]. Statins can lower coenzyme Q10 levels, and the National Lipid Association has endorsed coenzyme Q10 supplementation in a select group of patients who develop SAMS [47,182]. L-carnitine can be used in addition to coenzyme Q10 to help with SAMS [175]. Coenzyme Q10 is thought to reduce oxidative stress and replenish low coenzyme Q10 stores [44]. Typical dosages of coenzyme Q10 can range from 30 to 250 mg per day [45].

There are conflicting data on its lipid-lowering effects, which are minimal if present. In a meta-analysis of eight RCTs with 526 patients with known coronary artery disease, participants were randomized to placebo versus coenzyme Q10. In the arm with coenzyme Q10 supplementation, there was a decrease in TC (−0.03 mmol/L; 95% CI, −0.05 to −0.01) and an increase in HDL-C (0.03 mmol/L; 95% CI, 0.01 to 0.06). However, there were no significant changes in LDL-C, TG, or Lp(a) [46]. Conversely, a larger meta-analysis of 21 controlled trials with 1039 participants with metabolic syndrome found conflicting results. In the intervention arm, those being treated with coenzyme Q10 experienced a reduction in TG (−0.0032 mmol/L; 95% CI, −0.0063 to −0.0001). However, there were no significant changes in TC, HLD-C, or LDL-C [45].

In the Intervention With Selenium and Q10 on Cardiovascular Mortality and Cardiac Function in the Elderly Population in Sweden (KiSel-10) study, 443 Swedish participants between the ages of 70 to 87 years were randomized to receive placebo versus 200 mg coenzyme Q10 and 200 µg selenium supplementation for 4 years. Participants were followed for 12 years, and those in the supplement arm had a significant reduction in cardiovascular mortality compared to the placebo arm (HR 0.59; 95% CI, 0.42 to 0.81; *p* = 0.001) [183,184].

Coenzyme Q10 is generally well tolerated with no serious adverse events. Insomnia, gastrointestinal discomfort, dizziness, headache, dyspepsia, photophobia, irritability, and fatigue have been reported. Coenzyme Q10 may have interactions with anticoagulants, insulin, and certain cancer treatments. Beta-blockers can inhibit enzyme reactions involving coenzyme Q10 [47].

## 6. Lifestyle Changes and the Impact on Dyslipidemia

### The Mediterranean Diet

The Mediterranean diet mimics the food pattern typically consumed in areas surrounding the Mediterranean Sea. It can consist of plentiful fruits, vegetables, nuts, and grains. It can also comprise low to moderate amounts of dairy, poultry, fish, and eggs. The primary source of fat is derived from olive oil. Red or processed meats are less frequently consumed [185]. The Mediterranean diet is endorsed by the guidelines from multiple societies, including the European Society of Cardiology, European Association of Preventive Cardiology, American College of Cardiology, American Heart Association, and others [154,186].

In a meta-analysis of 38 studies with 4658 participants, those who had a Mediterranean diet showed a decrease in TC (−0.15 mmol/L; 95% CI, −0.26 to −0.04), TG (−0.14 mmol/L; 95% CI, −0.18 to −0.10), and LDL-C (−0.21 mmol/L; 95% CI, −0.35 to −0.08). There was also an increase in HDL-C (0.03 mmol/L; 95% CI, 0.01 to 0.06) [187].

In the Prevención con Dieta Mediterránea (PREDIMED) trial, 7447 participants from Spain with high cardiovascular risk were randomized into three arms, including Mediterranean diet supplemented with extra-virgin olive oil, Mediterranean diet supplemented with nuts, or a low-fat diet (control). Martınez-Gonzalez et al. followed the participants for about 5 years. Compared to the control arm, participants in the arms where their Mediterranean diets were supplemented with extra-virgin olive oil and nuts, respectively, experienced a 30% and 28% reduction in the combined endpoints of MI, stroke, and cardiovascular death. However, this was largely driven by the reduction in stroke, as there were no significant reductions in MI or cardiovascular death [188]. Martınez-Gonzalez et al. performed a post hoc analysis on the same PREDIMED cohort that focused on a “provegetarian” plant-based food pattern compared to those mostly consuming meat, fish, eggs, and dairy products. They found that those in the highest quintile for vegetable consumption had a 41% reduction in all-cause mortality compared to those with the lowest vegetable consumption [189].

Similarly, another meta-analysis of seven prospective cohort studies with 37,879 participants from Europe and the US also found a smaller but significant (15%) reduction in all-cause mortality in those following the Mediterranean diet. However, there was no significant reduction in cardiovascular mortality with the Mediterranean diet versus control. A subgroup analysis focusing on those consuming a Mediterranean diet also found that those living in the Mediterranean area had a more significant reduction in all-cause mortality compared to those from non-Mediterranean areas (14% versus 5%) [190].

## 7. Discussion

Nutraceuticals are a wide variety of compounds with various mechanisms of action, including inhibiting HMG-CoA reductase activity, inhibiting acyl-coenzyme A cholesterol acyltransferase, preventing cholesterol absorption from the gastrointestinal tract, promoting lipolysis, and more. The lipid-lowering effects of many nutraceuticals have been investigated in many rigorous clinical trials, systemic reviews, and meta-analyses. Many of these studies focused on primary prevention in patients with dyslipidemia, metabolic syndrome, and diabetes.

### 7.1. Comparisons of the Effectiveness of Nutraceuticals

Although a large amount of data regarding nutraceutical interventions exists, questions still remain regarding the comparative efficacy of nutraceuticals; this is attributed to the paucity of head-to-head trials comparing various nutraceuticals. A network meta-analysis (NMA) is considered the highest level of evidence-based medicine because it enables simultaneous comparison of interventions and provides indirect evidence of the comparative efficacy of various nutraceuticals [191].

A recent NMA by Osadnik et al. included 131 RCTs with over 13,062 participants and ranked the lipid-lowering effects of 10 nutraceuticals, including artichoke leaf extract, berberine, bergamot, garlic, green tea extract, policosanols, plant sterols and stanols, RYR extract, spirulina, and silymarin. Each nutraceutical was ranked according to *p*-score values. This NMA demonstrated that all analyzed nutraceuticals, except for policosanols in studies outside of Cuba, were more effective in lowering LDL-C than placebo [15].

Bergamot, RYR extract, artichoke, berberine, and plant sterols were ranked as the most effective in lowering LDL-C and TC by Osadnik et al. Bergamot was the most effective in lowering LDL-C and TC compared to the other nutraceuticals, and RYR extract was ranked as the second most effective supplement. Berberine and artichoke leaf extract were almost equally effective at reducing LDL-C and TC. In comparison with RYR and bergamot, berberine and artichoke leaf extract were ranked as slightly less effective.

Bergamot, berberine, and RYR extract were the three nutraceuticals that effectively raised HDL-C in the NMA performed by Osadnik et al. Bergamot was the most effective at raising HDL-C, followed by berberine and then RYR extract in terms of their effectiveness. Bergamot, RYR extract, silymarin, berberine, spirulina, artichoke leaf extract, and garlic were all shown to decrease TG levels, with bergamot being the most effect at lowering TG.

Osadnik et al. reported that the lipid-lowering effect of phytosterols was modest and ranked them fifth in their NMA. They might be more effective when combined with other nutraceuticals and exert a synergistic effect due to their various mechanisms of action. Phytosterols had the highest certainty of evidence as assessed by GRADE. Of note, Osadnik et al. reported that the results of Cuban trials on policosanols were questionable because similar results were not able to be reproduced in trials outside of Cuba. These disparate results reveal that more research is needed to investigate the true lipid-lowering potential of phytosterols [15].

### 7.2. Outcomes Data

RYR extract has the most data to support its use in patients with dyslipidemia. RYR extract supplementation is associated with a reduction in non-fatal MI, coronary revascularization, and sudden death [152]. These studies were primarily conducted in patients of Han Chinese descent, and caution should be taken when extrapolating to other patient populations.

Coenzyme Q10 can be used in patients who develop SAMS [182]. Despite the inconsistent data on the lipid-lowering efficacy, the KiSel-10 study demonstrated that Swedish patients between the ages of 70 to 87 years experienced a reduction in cardiovascular mortality when supplemented with both coenzyme Q10 and selenium [183,184]. Caution should be exercised when trying to extrapolate these results to other patient populations.

Mediterranean diets that are high in extra-virgin olive oil and nuts are the food patterns with the strongest evidence for reduction of MACE, due primarily to a reduction in stroke [188]. Similarly, the plant-based Mediterranean diet has also been shown to reduce all-cause mortality but currently is not shown to reduce cardiovascular mortality [189,190]. When examining those who follow the Mediterranean diet, those living in the Mediterranean had a higher reduction in all-cause mortality than those living outside of the Mediterranean, suggesting that other factors may be at play in reducing all-cause mortality [190].

Aged garlic extract has been shown to reduce the low-attenuation plaque percentage in patients with metabolic syndrome and DM2. These reductions were seen in serial imaging on cardiac computed tomography angiography after only 1 year of supplementation [156,157]. Further studies are needed to investigate the potential MACE reduction in patients taking aged garlic extract.

Niacin is no longer recommended for LDL-C reduction but may be considered for the treatment of hypertriglyceridemia in patients who are statin-intolerant [1,7]. Older studies have shown that niacin can reduce the risk of acute coronary syndrome, cerebral vascular accident, and revascularization in patients not on statins. Because statins were not used, those older studies do not reflect modern methodology and likely do not represent current-day patients receiving the standard-of-care [171]. The AIM-HIGH and HPS2-THRIVE trials confirmed this by showing that there was no benefit in the reduction of cardiovascular and cerebrovascular events when niacin was added on top of statin therapy [85,86]. The HPS2-THRIVE trial did show a reduction in coronary revascularization procedures [85].

Many nutraceuticals lack outcomes data. In turn, it should be highlighted that nutraceuticals do not replace the use of statins or detract from the importance of other lipid-lowering therapies. The controversial Supplements, Placebo or Rosuvastatin Study (SPORT) trial showed that rosuvastatin quickly lowered LDL-C more than either fish oil, cinnamon, garlic, turmeric, plant sterol, or red yeast rice extract over a 4 week period. The authors did not find a significant change in LDL-C with all six nutraceuticals compared to placebo. However, this study may have been underpowered, as it only had 190 participants among eight parallel arms in the follow-up period of 4 weeks [192].

### 7.3. Regulation of Nutraceuticals/Supplements

Nutraceutical preparations are often marketed as “food supplements”. Therefore, they are not regulated in the same manner as pharmaceuticals, so long as manufacturers do not make any specific health-related efficacy claims about their nutraceutical products. A common concern is the wide variation in quality, as there may be potential contaminants in formulations. There also may be differences in the amount of the actual marketed nutraceutical between different manufacturers or even within each single manufacturer due to batch-to-batch variations. Therefore, the results from one study may not be extrapolated to the exact nutraceutical formulation purchased by patients. Greater regulation is likely to result in the more widespread availability of high-quality preparations [14,15].

### 7.4. Future Research

There is a need for more high-quality RCTs, systematic reviews, and meta-analyses that investigate the lipid-lowering effects of nutraceuticals. Some nutraceutical studies are not as rigorous as pharmaceutical clinical trials, as they may have fewer patients enrolled. More work is needed to explore the impact of many nutraceuticals on biomarkers such as apoB, apolipoprotein A1, Lp(a), and others. In addition, more research is specifically needed to investigate the potential effects on plaque burden, MACE, and other outcomes. Furthermore, caution should be exercised when extrapolating the results to various ethnic groups or target populations with different medical conditions. Although many trials were run in primary prevention patients, more research is needed to investigate the use of nutraceuticals in secondary prevention. However, nutraceuticals may still serve an important role in the management of dyslipidemia, especially in patients with statin intolerance. They can also be used in patients who are interested in a more integrative approach with compounds that are extracted or purified from naturally occurring fruits, vegetables, plants, and meats. They can be added on top of statins and other conventional lipid-lowering therapies.

## 8. Conclusions

Bergamot and RYR extract appear to be the most effective nutraceuticals in terms of LDL-C reduction [15]. There are a wide variety of nutraceuticals that exhibit their lipid-lowering effects via different mechanisms of action [16,69]. A plant-based Mediterranean diet can also be incorporated into a patient’s lifestyle in order to provide a holistic approach to addressing dyslipidemia [187]. These integrative therapies may potentially help mitigate modifiable cardiovascular risk factors and potentially lower a patient’s ASCVD risk burden.

There is an ever-growing body of research on the lipid-lowering effects of nutraceuticals, as there are many clinical trials, systematic reviews, and meta-analyses. Interestingly, the SPORT trial has raised more questions about the potential lipid-lowering effects of nutraceuticals [192]. It is important to highlight that statins cannot be replaced by nutraceuticals, as statins remain the cornerstone of lipid-lowering therapy [16,69]. More studies are needed to investigate the lipid-lowering effects using robust modern clinical trial methodology involving statins.

Only a few integrative therapies have data on cardiovascular outcomes. Aged garlic extract has been shown to reduce low-attenuation plaque [156,157]. However, only RYR extract, coenzyme Q10 (when supplemented with selenium), and the Mediterranean diet have been shown to have some mortality benefits [152,183,184,188]. Hence, more research is needed to investigate the impact of other nutraceuticals on cardiovascular risk on a long-term basis using modern clinical research methodology.

Nutraceuticals used to treat dyslipidemia have been gaining popularity with both patients and clinicians [12]. They can be used in patients who have SAMS or other side effects from statins. They can be considered in patients with dyslipidemia but who are ineligible for statin therapy. Nutraceuticals can also be used when patients have a strong preference over conventional therapies. They can be used as the initial therapy or as an adjunct therapy on top of pharmaceuticals or other nutraceuticals [14,16,69]. Since many nutraceuticals come from all around the world, some patients may prefer nutraceuticals for cultural, ritualistic, or religious reasons. It is important that clinicians be able to provide culturally competent care that aligns with their patients’ values and beliefs.

Integrative cardiologists are uniquely equipped to guide this emerging field of medicine and the use of nutraceuticals in the management of dyslipidemia. There is a potential opportunity to reduce ASCVD risk and to promote cardiovascular prevention [12]. Clinicians can help patients obtain accurate information and continue to generate the science to support the clinical use of nutraceuticals. There is a lot of exciting work to be done to further explore the evolving story of lipid management.

## Figures and Tables

**Table 1:** Nutraceuticals and their LDL-Lowering Effects.

Nutraceutical	Class	Level of Evidence	Mechanism of Action	Daily Dose	Lipid Lowering Effect	Safety	Drug Interactions
Anthocyanins	IIb	A	Downregulates the mRNA of SREBP-1c and fatty acid synthase, resulting in less fat accumulation in adipocytes [17]	100 to 450 mg [18]	LDL-C: −5 to −10% [16]	Well tolerated [16] Precautions during pregnancy (polyhydramnios) [19]	No known drug interactions [16]
Artichoke Leaf Extract	IIa	A	Inhibit HMG-CoA reductase [16,20], induce pathways involving SREBP and ACAT, [20] increase excretion of bile acids [21]	500 to 2800 mg [21]	LDL-C: −5 to −15% [16]	GI discomfort, skin reactions, and asthma exacerbations [16,22] Insufficient data in pregnant or breastfeeding patients [22]	Antidiabetic drugs, antihypertensive drugs, and CYP2B6 and CPY2C19 inducers and inhibitors [22]
Berberine	I	A	PCSK9-I [23], upregulates the hepatic LDL receptor by activing the Jun amino-terminal kinase and extracellular signal regulated kinases [16,24] Decreased GI reabsorption of cholesterol, enhanced fecal excretion, increased hepatic bile acid formation, [25] activates AMPK, [26,27] inhibits NADPH [28]	200 to 1500 mg [29]	LDL-C: −15 to −20% [16]	GI symptoms, headaches, and elevated transaminases [30,31] No serious liver injury [16,29,31] Kernicterus in infants [31] Pregnant women, breastfeeding mothers, and infants should avoid [22]	Anticoagulants, anti- platelets, antidiabetic drugs, antihypertensive drugs, central nervous system depressants, cyclosporine, CYP substrates (CYP2C9, CYP2D6, CYP3A4), dextromethorphan, and tacrolimus [22]
Bergamot	IIa	A	Inhibits HMG-CoA reductase, ACAT [16] pancreatic cholesterol ester hydrolase, [32] activates AMPK, [26] radical-scavenging activity, [16] increases fecal excretion of bile acids [16,33]	200 to 1500 mg [16]	LDL-C: −7 to −40% [34]	GI discomfort and muscle cramps Little safety data in patients who are pregnant or breastfeeding [22]	Antidiabetic drugs due to hypoglycemia [22]
L-carnitine	IIb	A	Decreases TG synthesis by decreasing available free fatty acids, increases mitochondrial oxidation of long chain fatty acids, and increases production of apolipoprotein A1 [35]	500 mg to 6 g [35]	LDL-C: −0.14 mmol/L; 95% CI, –0.22 to −0.06 TG: −0.11 mmol/L; 95% CI, –0.18 to −0.03 [35]	Fishy body odor, minor nausea, or GI discomfort [22,36] Safe in patients who are pregnant or breastfeeding Parenteral carnitine supplementation is safe in infants [22,37,38]	Thyroid hormones and warfarin [22]
Chromium	III	A	Upregulates gene expression of PPAR-γ and LDL receptor [39]	40 to 1000 mcg [39]	TC: −0.17 mmol/L; 95% CI, –0.27 to −0.07 [40]	Weight loss, hypoglycemia, anemia, thrombocytopenia, elevated transaminases, elevated creatinine, rhabdomyolysis, and dermatitis Safe during pregnancy and in women breastfeeding [41]	Levothyroxine, insulin, metformin, and other anti- diabetic medication [41]
Chitosan	IIb	A	Interferes with GI absorption by binding to negatively charged fatty acids and bile acids and disrupting the emulsification of neutrally charged cholesterol [42]	0.3 to 3 g [43]	LDL-C: −5% [16]	Should not be used in patients with allergies to crustaceans or shellfish [42] GI discomfort Insufficient data on use in patients who are pregnant or breastfeeding [22]	Acyclovir or warfarin [22]
Coenzyme Q10	III	A	Reduce oxidative stress and restores coenzyme Q10 [44]	30 to 250 mg [45]	TG: −0.0032 mmol/L; 95% CI, –0.0063 to −0.0001 [45] HDL-C: 0.03 mmol/L; 95% CI, 0.01 to 0.06 [46]	Insomnia, GI discomfort, dizziness, headache, dyspepsia, photophobia, irritability, and fatigue [47]	Anticoagulants, insulin, and cancer treatments Beta-blockers can inhibit enzyme reactions involving coenzyme Q10 [47]
Conjugated Linoleic Acid	IIb	A	Promotes cholesterol efflux by increasing expression of ABCA1 and ABCG1 [48]	0.5 to 7 g [49]	LDL-C: −5% [16]	GI discomfort, hepatotoxicity [50] Safe in patients who are pregnant [51] Insufficient data in patients breastfeeding [22]	Anticoagulants, anti- platelet drugs, and antihypertensives [22]
Curcumin	IIb	A	Inhibits intestinal NPC1L1 cholesterol transporter expression by inhibiting the SREBP2 transcription factor, [52] downregulates PCSK9 expression, [53] upregulates ABCA1 expression [54]	50 mg to 6 g [55]	LDL-C: −5% [16]	GI discomfort [22,56] Precautions in patients who are pregnant or breastfeeding as levels greater than dietary levels may be unsafe [22]	Alkylating agents, amlodipine, anticoagulants, anti- platelets, antidiabetic drugs, CYP3A4 substrates, sulfasalazine, tacrolimus, talinolol, tamoxifen, and warfarin [22]
Soluble Fiber							
Glucomannan	IIa	A	Inhibits HMG-CoA reductase, reduces GI cholesterol absorption, [57] increases the conversion of bile acids into cholesterol through increased 7-α- hydroxylase activity [16]	1 to 15 g per day [16,58]	LDL: −0.35 mmol/L; 95% CI, –0.46 to −0.25 [58]	GI discomfort, obstructions in the bowel or esophagus, [22,58] reduce absorption of vitamin E Insufficient data in patients who are pregnant or breastfeeding [22]	May be issues with absorption of medicines [22]
β-Glucan	IIa	A	Viscosity reduces cholesterol absorption, increases bile acid excretion [59]	3 to 25 g [16,60]	LDL-C: −0.27 mmol/L; 95% CI, −0.35 to −0.20 [60]	Insufficient data in patients who are pregnant or breastfeeding [22]	antihypertensives and immunosuppressant drugs [22]
Guar Gum	IIa	A	Prevents GI cholesterol absorption, increases bile acid extraction [61]	30 to 100 g [61]	LDL-C: −0.45 mmol/L; 95% CI, –0.61 to −0.29 [61]	GI discomfort, obstruction of the esophagus or bowel [22,61] Safe during pregnancy as it treats intrahepatic cholestasis of pregnancy [62] Insufficient data in patients who are breastfeeding [22]	Penicillin, metformin, estradiol, and digoxin May inhibit the absorption of oral drugs [22]
Psyllium	IIa	A	Reduces GI absorption cholesterol, binds to bile acids [63]	2 to 20 g per day [63]	LDL-C: −0.33 mmol/L; 95% CI, –0.38 to −0.27 [63]	GI discomfort, mild anaphylactic allergic reactions, bowel obstructions, and esophageal obstructions [16,22,64] Safe during pregnancy or while breastfeeding [65]	Carbamazepine, digoxin, estradiol, lithium, metformin, and olanzapine [22]
							
Garlic Extract	IIa	A	Inhibits HMG-CoA reductase, acetyl-CoA synthetase, squalene- monooxygenase, and potentially non- acetylated CoA, [66] GI absorption of fatty acids and cholesterol, increases the excretion of bile acids [16]	0.3 to 20 g [16,67]	LCL-C: −5 to −10% [16]	GI symptoms, body odor, garlicy breath, aftertaste, and increased bleeding risk [16,68] Possibly unsafe when used in higher levels in patients who are pregnant or breastfeeding [22]	Anti-hypertensive drugs, anti-diabetic drugs, atazanavir, CYP2E1/CYP3A4 inducers and inhibitors, isoniazid, protease inhibitors, saquinavir, tacrolimus, and warfarin [22]
Green Tea	IIa	A	Inhibits expression of nitric oxide synthase, [69] activates AMPK, inhibits HMG-CoA reductase, [16,69] inhibits the reabsorption of bile acids [16,70]	100 mg to 20 g per day [16,69]	LCL-C: −5 [16]	GI discomfort, transitory blood pressure elevations, rashes thrombotic thrombocytopenic purpura, hepatotoxicity, hypokalemia, [16,22,69] Higher doses associated with iron and folate deficiency; use with precaution in pregnant patients [16,71]	5-fluorouracil, adenosine, anti- coagulants, anti- platelets, antidiabetic drugs, statins, beta agonists, bortezomib, carbamazepine, cimetidine, clozapine, lisinopril, lithium, stimulants, verapamil, and valproate acid [22]
Alpha Lipoic Acid	IIb	A	Modulates fat synthesis, mitochondrial β- oxidation of fat, clearance of TG-rich lipoproteins in the liver, and adipose TG accumulation [72]	300 to 1800 mg [73]	LDL-C: −0.28 mmol/L; 95% CI, −0.50 to −0.06 [73]	GI discomfort, skin rash, and rarely insulin autoimmune syndrome Safe during pregnancy [74] Insufficient data in patients breastfeeding [22]	Alkylating agents, anticoagulants, anti- platelet drugs, antidiabetic drugs, antitumor antibiotics, and levothyroxine [22]
Lupin Protein	IIa	A	Inhibits HMG-CoA receptor and PCSK9 activity. Refs. [75,76], upregulates SREBP-2 via phosphatidylinositol-3- kinase, alpha serine/threonine-protein kinase, and glycogen synthase kinase-3 beta kinase pathways [77]	≤35 g [78]	LDL-C: −5 to −12% [16]	GI discomfort Likely safe to use in patients who are pregnant or breastfeeding [22]	No known interactions with drugs [22]
Magnesium	III	A	Regulates HMG-CoA reductase expression, upregulates cholesterol 7α-hydroxylase and LCAT expression [79]	35 to 500 mg [80]	LDL-C: −0.18 mmol/L; 95% CI, –0.30 to −0.05 [81]	GI discomfort, flushing, confusion, hypotension, hyperreflexia, respiratory depression, hyperkalemia, hypocalcemia, pulmonary edema, and cardiac arrest [82,83] Safe during pregnancy at appropriate doses [22,83] Appears safe to use during breastfeeding [22]	Aminoglycosides, antacids, bisphosphonates, calcium channel blockers, digoxin, ketamine, levodopa, carbidopa, potassium-sparing diuretics, quinolones, sulfonylurea, and tetracyclines [22]
Niacin	IIb	A	Inhibits diacylglycerol acyltransferase-2, which decreases TG synthesis and LDL-C by increasing hepatic apoB degradation, raises HDL-C by stimulating hepatic apolipoprotein A-I production [84]	≤2 g daily [85,86]	TG: −28.6%; LDL-C: −12.0% [86]	GI hemorrhage, peptic ulcers, myopathy, rhabdomyolysis, gout, flushing, skin lesions, skin infections, lower respiratory infections, and increased incidence of diabetes and hospitalizations for diabetes [85] No restrictions for pregnant or breast feeding patients [87]	Statins, isoniazid, and pyrazinamide [1,88]
Nigella Sativa	IIb	A	Reduces GI cholesterol absorption, increases biliary excretion, reduces cholesterol synthesis, inhibits lipid oxidation, upregulates LDL receptors [89]	200 mg to 3 g for powders, capsules, and extracts 1 to 5 mL for oil suspensio ns [90]	LDL-C: −0.48 mmol/L; 95% CI, –0.58 to −0.37 [90]	GI discomfort, elevated alkaline-phosphate, AST, ALT, gamma- glutamyl transferase [89] Safe to use during pregnancy [91] Insufficient data during breastfeeding [22]	anticoagulants, anti- platelets, antidiabetic drugs, antihypertensives, cyclosporine, diuretics, immunosuppressants, and serotonergic drugs [22]
Olive Extract	IIb	B	Reduces lipid peroxidation, increases bile excretion, and inhibits HMG-CoA reductase and ACAT [92]	136.2 mg oleuropei n and 6.4 mg hydroxyty rosol [93]	LDL-C: –0.19 ± SD 0.56 mmol/L [93]	No known adverse effects of olive extract No known data on about the levels consumed through olive extract in those who are pregnant or breastfeeding [94]	No known interactions [94]
Gamma-oryzanol	IIb	A	Inhibits GI absorption, increases excretion of bile acids, inhibits HMG- CoA reductase, inhibits platelet aggregation, [95] alters the gut microbiome [96]	100 to 300 mg [16,22]	LDL-C: −5 to −10% [16]	No reported side effects [10,97] No safety data on patients who are pregnant or breastfeeding [22]	No known drug interactions [22]
Pantethine	IIb	B	Inhibits HMG-CoA reductase and acetyl- CoA carboxylase, which are involved in TG synthesis and lipoprotein metabolism [98]	600 to 1200 mg [98]	LDL-C: −11% [98]	gastrointestinal symptom [98] Safe in children and in patients with chronic kidney disease, including dialysis [99,100] Insufficient data in patients who are pregnant or breastfeeding [22]	No known drug interactions [22]
Polyunsaturated n-3 Fatty Acids	I	A	Reduces synthesis of hepatic VLDL, endogenous fatty acids, substrates available for TG synthesis, and activity of diacylglycerol acyltransferase or phosphatidic acid phosphohydrolase, which are involved in TG synthesis, promotes β- oxidation of fatty acids and increase phospholipid synthesis [101]	≤4 g [102,103]	TG: −0.36 mmol/L [104]	GI discomfort, new- onset atrial fibrillation and atrial flutter, fishy aftertaste [103,105] Safe to use in pregnancy [106] Likely safe during breastfeeding [22]	Anticoagulants, anti- platelet drugs, antihypertensives, contraceptives, cyclosporine, and tacrolimus [22]
Pectin	IIb	B	Decreases GI cholesterol absorption, increases bile acid excretion, [107,108] decreases HMG-CoA reductase, increases cholesterol 7-α- hydroxylase in the liver, modulates gut microbiome [108]	6 g [107]	LDL-C: −5 to −10% [16]	GI discomfort [107] Precaution in patients who are pregnant (binds to vitamin B12) [109] Safe in patients who are breastfeeding [22]	Digoxin, lovastatin, and tetracyclines [22]
Phytosterols	IIa	A	Inhibit cholesterol absorption in GI tract by competing with dietary cholesterol in the formation of dietary micelles, decreasing apoB secretion from enterocytes and hepatocytes, increases expression of ABCA1 and ABCG1 level [110,111,112]	400 mg to 3 g [16,110]	LDL-C: −8 to −16% [16,69]	Well tolerated and safe [16,69] Safe to use in pregnancy [113] Insufficient data on their use in those breastfeeding [22]	No known drug interactions [22]
Policosanol	IIb	A	Promotes bile acid production and lipolysis via inhibiting the expression of farnesoid X receptor-small heterodimer partner and activating the Takeda G- coupled protein receptor 5-AMPK signaling pathway, which inhibits HMG-CoA reductase activity [114,115]	5 to 20 mg [116]	LDL-C: −0.71 mmol/L; 95% CI, –1.02 to −0.40 [116]	GI discomfort, tachycardia, myalgia, hypertension, headache, dizziness, somnolence, insomnia, polydipsia, nocturia, dry skin, rashes, and weight gain [116] Safe to use in pregnancy [117,118] Insufficient data in breastfeeding [22]	Antidiabetic drugs, beta-blockers, nitroprusside, and warfarin [22]
Probiotics	IIb	A	*Lactobacillus* and *Bifidobacterium* increase bile acid excretion [119] *Lacticaseibacillus* decreases NPC1L1 cholesterol transporter expression and increase cholesterol efflux via increased expression of ABCA1 [120] *Lactobacillus rhamnosus* JL1 activates the AMPK pathway and inhibits PPAR-γ and SREBP-1C gene expression [121]	1 to 6 g [16,122]	LDL-C: −5% [16]	GI discomfort, infections from yeast *Saccharomyces cervisiae* Safe in infants and patients who are pregnant or breastfeeding [123]	Insufficient data [16]
Red Yeast Rice Extract	I	A	HMG-CoA reductase inhibitor [124]	<3 mg of monacolin K) [14]	LDL-C: −15% to −25% [16]	Gastrointestinal discomfort, rashes, and allergic reactions [17,20,21] Formulations with citrinin are nephrotoxic and hepatotoxic [14,20,21] No elevations in transaminases or kidney impairment [14,17,20,21] Precautions with pregnancy [125] Unknown if safe to use during breastfeeding [126]	CYPP450 inducers or inhibitors, antifungals, macrolides, cyclosporine, fibrates, niacin, nefazodone, protease inhibitors, statins, and verapamil [16,126]
Resveratrol	III	A	Activates silent information regulation 2 homolog 1, suppresses hepatic upregulation of genes associated in lipogenesis and prevent the downregulation of genes involved with lipolysis, [127] suppresses foam cell formation [128]	250 to 3000 mg [129,130]	LDL-C: −0.147 mmol/L; 95% CI, –0.286 to −0.008 [131]	Increase bleeding Likely safe to consume during pregnancy and breastfeeding provided not consumed from alcohol. In patients with malignancies that grow in response to estrogen, such as breast cancer, uterine cancer, ovarian cancer, it is advised that patients should limit intake [129]	Garlic, ginger, ginkgo, nattokinase, anticoagulants, anti- platelet drugs and those involving the cytochrome P450 system, such as CYP1A1, CYP1A2, CYP1B1, CYP2C19, CYP2E1, and CYP3A4 [129]
Sea Buckthorn	IIb	A	Increases expression of PPAR-α, PPAR-γ, and ABCA1, decreases expression of SREBP-2, promotes expression of CPT1A, which is involved in increasing lipolysis and β-oxidation [132]	0.75 mL [133]	LDL-C: −0.40 mmol/L; 95% CI, –0.76 to −0.04 [133]	Well tolerated Safe to use in pregnancy [134] Little data in patients who breastfeed [22]	Anticoagulants, anti- platelets, and antihypertensives [22]
Silymarin	IIb	A	Increases lipolysis and β- oxidation via the upregulation of CPT1, [135] increases cholesterol efflux via increases expression of ABCA1 [136]	140 to 700 mg [137]	LDL-C: −0.27 mmol/L; 95% CI, –0.49 to −0.05 [137]	GI discomfort, headache, ureteric calculi, and hemolytic anemia, transient ischemia attack, myocardial infarction, and cardiovascular death [137] Insufficient data in patients who are pregnant or breastfeeding [138]	Antidiabetic drugs, morphine, tamoxifen, sirolimus, and warfarin [22]
Spirulina	IIa	A	Activates heme oxygenase-1 (atheroprotective enzyme involved in heme catabolic pathway in endothelial cells), [16,69] antioxidant, anti- inflammatory, and radical-scavenging properties, [16] alters gut microbiome [139]	1 to 10 g [69]	LDL-C: −5 to −15% [16,69]	GI discomfort, bleeding, rashes, elevated transaminases, and cholestasis [22,140] Little safety data in patients who are pregnant or breastfeeding [22]	Anticoagulants, anti- platelets, antidiabetic drugs, and immunosuppressant drugs [22]
Soybean Protein	IIa	A	Inhibits HMG-CoA reductase, reduces PCSK9 protein, [141] increases bile acid excretion, cholesterol synthesis inhibition, increases transcription of the LDL receptor, [142,143] increases apoB receptor activity, decreases hepatic synthesis of cholesterol and lipoprotein secretion, [143] alters the gut microbiome [144]	25 to 120 mg [16,69]	LDL-C: −3 to −10% [16]	GI discomfort, menstrual complaints, headaches, dizziness, and rashes [22] Chronic use of higher doses of soy protein may affect fertility and thyroid function [16,69] Decreased absorption of divalent and trivalent metals, such as calcium, copper, iron, magnesium, and zinc [143] Avoid during pregnancy Use of soy is likely safe during breastfeeding [22]	Antidiabetic drugs, antihypertensive drugs, diuretics, estrogens, progesterone, tamoxifen, levothyroxine, monoamine oxidase inhibitors, and warfarin [22]
Vitamin E	IIb	A	Inhibits HMG-CoA reductase, promotes scavenging for free radicals, and activates PPAR -α, -β, and -γ receptors [145,146,147]	400 to 800 UI [16]	HDL-C: 0.15 mmol/L; 95% CI, 0.07 to 0.23 [148]	Bleeding, heart failure, hemorrhagic cerebral vascular accidents, prostate cancer, and all- cause mortality [147,149] Safe in pregnancy [106] Likely safe during breastfeeding [22]	Alkylating agents, anticoagulants, anti- platelets, cyclosporine, CYP3A4 substrates, niacin, and selumetinib [22]

Definition of level of evidence. Level A: Data derived from multiple randomized clinical trials or their meta-analysis. Level B: Data derived from a single randomized clinical trial or large nonrandomized studies. Level C: Consensus or opinion of experts and/or small studies, retrospectives studies, or registries. Definition of classes of recommendation. Class I: Evidence and/or general agreement that a given treatment or procedure is beneficial, useful, and effective. Is recommended/ is indicated. Class II: Conflicting evidence and/or a divergence of opinion about the usefulness/efficacy of the given treatment or procedure. Class IIa: Weight of evidence/opinion is in favor of usefulness/ efficacy. Should be considered. Class IIb: Usefulness/efficacy is less well established by evidence/opinion. May be considered. Class III Evidence or general agreement that the given treatment or procedure is not useful/ effective and, in some cases, may be harmful. Is not recommended (no efficacy on lipid profile). Abbreviations: Acyl-coenzyme A cholesterol acyltransferase (ACAT), adenosine triphosphate-binding cassette (ABC), alanine-aminotransferase (ALA), adenosine monophosphate-activated protein kinase (AMPK), aspartate-aminotransferase (AST), carnitine palmitoyl transferase 1 (CPT1), gastrointestinal reabsorption (GI), high-density lipoprotein cholesterol (HDL-C), 3-hydroxy-3-methylglutaryl-coenzyme A (HMG-CoA), lecithin cholesterol acyltransferase (LCAT), low-density lipoprotein cholesterol (LDL-C), messenger ribonucleic acid (mRNA), nicotinamide adenine dinucleotide phosphate (NADPH), Niemann-Pick C1-like 1 (NPC1L1), peroxisome proliferator-activated receptor (PPAR), proprotein convertase subtilisin/kexin type 9 inhibitor (PCSK9I), red yeast rice (RYR), sterol regulatory element-binding proteins (SREBP), total cholesterol (TC), triglycerides (TG).

## Data Availability

Not applicable.

## References

[1] Grundy S.M., Stone N.J., Bailey A.L., Beam C., Birtcher K.K., Blumenthal R.S., Braun L.T., de Ferranti S., Faiella-Tommasino J., Forman D.E. (2019). 2018 aha/acc/aacvpr/aapa/abc/acpm/ada/ags/apha/aspc/nla/pcna guideline on the management of blood cholesterol: A report of the american college of cardiology/american heart association task force on clinical practice guidelines. J. Am. Coll. Cardiol..

[2] Ambrosino P., Bachetti T., D’Anna S.E., Galloway B., Bianco A., D’Agnano V., Papa A., Motta A., Perrotta F., Maniscalco M. (2022). Mechanisms and clinical implications of endothelial dysfunction in arterial hypertension. J. Cardiovasc. Dev. Dis..

[3] Mućka S., Miodońska M., Jakubiak G.K., Starzak M., Cieślar G., Stanek A. (2022). Endothelial function assessment by flow-mediated dilation method: A valuable tool in the evaluation of the cardiovascular system. Int. J. Env. Res. Public Health.

[4] Jakubiak G.K., Cieślar G., Stanek A. (2022). Nitrotyrosine, nitrated lipoproteins, and cardiovascular dysfunction in patients with type 2 diabetes: What do we know and what remains to be explained?. Antioxidants.

[5] Raised Cholesterol. https://www.who.int/data/gho/indicator-metadata-registry/imr-details/3236.

[6] Ference B.A., Ginsberg H.N., Graham I., Ray K.K., Packard C.J., Bruckert E., Hegele R.A., Krauss R.M., Raal F.J., Schunkert H. (2017). Low-density lipoproteins cause atherosclerotic cardiovascular disease. 1. Evidence from genetic, epidemiologic, and clinical studies. A consensus statement from the european atherosclerosis society consensus panel. Eur. Heart J..

[7] Mach F., Baigent C., Catapano A.L., Koskinas K.C., Casula M., Badimon L., Chapman M.J., De Backer G.G., Delgado V., Ference B.A. (2020). 2019 esc/eas guidelines for the management of dyslipidaemias: Lipid modification to reduce cardiovascular risk. Eur. Heart J..

[8] Gidding S.S., Champagne M.A., de Ferranti S.D., Defesche J., Ito M.K., Knowles J.W., McCrindle B., Raal F., Rader D., Santos R.D. (2015). The agenda for familial hypercholesterolemia: A scientific statement from the american heart association. Circulation.

[9] Grundy S.M., Stone N.J., Guideline G. (2019). Writing Committee for the Cholesterol. 2018 cholesterol clinical practice guidelines: Synopsis of the 2018 american heart association/american college of cardiology/multisociety cholesterol guideline. Ann. Intern Med..

[10] Cheeley M.K., Saseen J.J., Agarwala A., Ravilla S., Ciffone N., Jacobson T.A., Dixon D.L., Maki K.C. (2022). Nla scientific statement on statin intolerance: A new definition and key considerations for ascvd risk reduction in the statin intolerant patient. J. Clin. Lipidol..

[11] Bytyci I., Penson P.E., Mikhailidis D.P., Wong N.D., Hernandez A.V., Sahebkar A., Thompson P.D., Mazidi M., Rysz J., Pella D. (2022). Prevalence of statin intolerance: A meta-analysis. Eur. Heart J..

[12] Cheung B. (2022). The intersection of the two complimentary fields of preventive cardiology and integrative cardiology: A fellow’s voice. Am. J. Prev. Cardiol..

[13] Bin Y.S., Kiat H. (2011). Prevalence of dietary supplement use in patients with proven or suspected cardiovascular disease. Evid.-Based Complement. Alternat. Med..

[14] Banach M., Catapano A.L., Cicero A.F.G., Escobar C., Foger B., Katsiki N., Latkovskis G., Rakowski M., Reiner Z., Sahebkar A. (2022). Red yeast rice for dyslipidaemias and cardiovascular risk reduction: A position paper of the international lipid expert panel. Pharmacol. Res..

[15] Osadnik T., Golawski M., Lewandowski P., Morze J., Osadnik K., Pawlas N., Lejawa M., Jakubiak G.K., Mazur A., Schwingschackl L. (2022). A network meta-analysis on the comparative effect of nutraceuticals on lipid profile in adults. Pharmacol. Res..

[16] Cicero A.F.G., Colletti A., Bajraktari G., Descamps O., Djuric D.M., Ezhov M., Fras Z., Katsiki N., Langlois M., Latkovskis G. (2017). Lipid-lowering nutraceuticals in clinical practice: Position paper from an international lipid expert panel. Nutr. Rev..

[17] Park S., Kang S., Jeong D.Y., Jeong S.Y., Park J.J., Yun H.S. (2015). Cyanidin and malvidin in aqueous extracts of black carrots fermented with aspergillus oryzae prevent the impairment of energy, lipid and glucose metabolism in estrogen-deficient rats by ampk activation. Genes Nutr..

[18] Araki R., Yada A., Ueda H., Tominaga K., Isoda H. (2021). Differences in the effects of anthocyanin supplementation on glucose and lipid metabolism according to the structure of the main anthocyanin: A meta-analysis of randomized controlled trials. Nutrients.

[19] Kapadia V., Embers D., Wells E., Lemler M., Rosenfeld C.R. (2010). Prenatal closure of the ductus arteriosus and maternal ingestion of anthocyanins. J. Perinatol..

[20] Rangboo V., Noroozi M., Zavoshy R., Rezadoost S.A., Mohammadpoorasl A. (2016). The effect of artichoke leaf extract on alanine aminotransferase and aspartate aminotransferase in the patients with nonalcoholic steatohepatitis. Int. J. Hepatol..

[21] Sahebkar A., Pirro M., Banach M., Mikhailidis D.P., Atkin S.L., Ciceo A.F.G. (2018). Lipid-lowering activity of artichoke extracts: A systematic review and meta-analysis. Crit. Rev. Food Sci. Nutr..

[22] (2022). Natural Medicines. https://naturalmedicines.therapeuticresearch.com/.

[23] Li H., Dong B., Park S.W., Lee H.S., Chen W., Liu J. (2009). Hepatocyte nuclear factor 1alpha plays a critical role in pcsk9 gene transcription and regulation by the natural hypocholesterolemic compound berberine. J. Biol. Chem..

[24] Abidi P., Zhou Y., Jiang J.D., Liu J. (2005). Extracellular signal-regulated kinase-dependent stabilization of hepatic low-density lipoprotein receptor mrna by herbal medicine berberine. Arter. Thromb. Vasc. Biol..

[25] Li X.Y., Zhao Z.X., Huang M., Feng R., He C.Y., Ma C., Luo S.H., Fu J., Wen B.Y., Ren L. (2015). Effect of berberine on promoting the excretion of cholesterol in high-fat diet-induced hyperlipidemic hamsters. J. Transl. Med..

[26] Loh K., Tam S., Murray-Segal L., Huynh K., Meikle P.J., Scott J.W., van Denderen B., Chen Z., Steel R., LeBlond N.D. (2019). Inhibition of adenosine monophosphate-activated protein kinase-3-hydroxy-3-methylglutaryl coenzyme a reductase signaling leads to hypercholesterolemia and promotes hepatic steatosis and insulin resistance. Hepatol. Commun..

[27] Kim W.S., Lee Y.S., Cha S.H., Jeong H.W., Choe S.S., Lee M.R., Oh G.T., Park H.S., Lee K.U., Lane M.D. (2009). Berberine improves lipid dysregulation in obesity by controlling central and peripheral ampk activity. Am. J. Physiol. Endocrinol. Metab..

[28] Wang C., Li J., Lv X., Zhang M., Song Y., Chen L., Liu Y. (2009). Ameliorative effect of berberine on endothelial dysfunction in diabetic rats induced by high-fat diet and streptozotocin. Eur. J. Pharmacol..

[29] Bertuccioli A., Moricoli S., Amatori S., Rocchi M.B.L., Vici G., Sisti D. (2020). Berberine and dyslipidemia: Different applications and biopharmaceutical formulations without statin-like molecules-a meta-analysis. J. Med. Food.

[30] Ju J., Li J., Lin Q., Xu H. (2018). Efficacy and safety of berberine for dyslipidaemias: A systematic review and meta-analysis of randomized clinical trials. Phytomedicine.

[31] Linn Y.C., Lu J., Lim L.C., Sun H., Sun J., Zhou Y., Ng H.S. (2012). Berberine-induced haemolysis revisited: Safety of rhizoma coptidis and cortex phellodendri in chronic haematological diseases. Phytother. Res..

[32] Musolino V., Gliozzi M., Carresi C., Maiuolo J., Mollace R., Bosco F., Scarano F., Scicchitano M., Maretta A., Palma E. (2017). Lipid-lowering effect of bergamot polyphenolic fraction: Role of pancreatic cholesterol ester hydrolase. J. Biol. Regul. Homeost. Agents.

[33] Cappello A.R., Dolce V., Iacopetta D., Martello M., Fiorillo M., Curcio R., Muto L., Dhanyalayam D. (2016). Bergamot (*Citrus bergamia* risso) flavonoids and their potential benefits in human hyperlipidemia and atherosclerosis: An overview. Mini Rev. Med. Chem..

[34] Lamiquiz-Moneo I., Gine-Gonzalez J., Alisente S., Bea A.M., Perez-Calahorra S., Marco-Benedi V., Baila-Rueda L., Jarauta E., Cenarro A., Civeira F. (2020). Effect of bergamot on lipid profile in humans: A systematic review. Crit. Rev. Food Sci. Nutr..

[35] Askarpour M., Hadi A., Symonds M.E., Miraghajani M., Sadeghi O., Sheikhi A., Ghaedi E. (2019). Efficacy of l-carnitine supplementation for management of blood lipids: A systematic review and dose-response meta-analysis of randomized controlled trials. Nutr. Metab. Cardiovasc. Dis..

[36] Cruciani R.A., Dvorkin E., Homel P., Malamud S., Culliney B., Lapin J., Portenoy R.K., Esteban-Cruciani N. (2006). Safety, tolerability and symptom outcomes associated with l-carnitine supplementation in patients with cancer, fatigue, and carnitine deficiency: A phase i/ii study. J. Pain Symptom. Manag..

[37] Seong S.H., Cho S.C., Park Y., Cha Y.S. (2010). L-carnitine-supplemented parenteral nutrition improves fat metabolism but fails to support compensatory growth in premature korean infants. Nutr. Res..

[38] Keller U., Van Der Wal C., Seliger G., Scheler C., Röpke F., Eder K. (2009). Carnitine status of pregnant women: Effect of carnitine supplementation and correlation between iron status and plasma carnitine concentration. Eur. J. Clin. Nutr..

[39] Tarrahi M.J., Tarrahi M.A., Rafiee M., Mansourian M. (2021). The effects of chromium supplementation on lipid profile in humans: A systematic review and meta-analysis of randomized controlled trials. Pharmacol. Res..

[40] Asbaghi O., Naeini F., Ashtary-Larky D., Moradi S., Zakeri N., Eslampour E., Kelishadi M.R., Naeini A.A. (2021). Effects of chromium supplementation on lipid profile in patients with type 2 diabetes: A systematic review and dose-response meta-analysis of randomized controlled trials. J. Trace Elem. Med. Biol..

[41] (2022). Chromium Fact Sheet for Health Professionals. NIH. https://ods.od.nih.gov/factsheets/Chromium-HealthProfessional/#h2.

[42] Ylitalo R., Lehtinen S., Wuolijoki E., Ylitalo P., Lehtimäki T. (2002). Cholesterol-lowering properties and safety of chitosan. Arzneimittelforschung.

[43] Moraru C., Mincea M.M., Frandes M., Timar B., Ostafe V. (2018). A meta-analysis on randomised controlled clinical trials evaluating the effect of the dietary supplement chitosan on weight loss, lipid parameters and blood pressure. Medicina.

[44] Arenas-Jal M., Sune-Negre J.M., Garcia-Montoya E. (2020). Coenzyme q10 supplementation: Efficacy, safety, and formulation challenges. Compr. Rev. Food Sci. Food Saf..

[45] Sharifi N., Tabrizi R., Moosazadeh M., Mirhosseini N., Lankarani K.B., Akbari M., Chamani M., Kolahdooz F., Asemi Z. (2018). The effects of coenzyme q10 supplementation on lipid profiles among patients with metabolic diseases: A systematic review and meta-analysis of randomized controlled trials. Curr. Pharmacol. Des..

[46] Jorat M.V., Tabrizi R., Mirhosseini N., Lankarani K.B., Akbari M., Heydari S.T., Mottaghi R., Asemi Z. (2018). The effects of coenzyme q10 supplementation on lipid profiles among patients with coronary artery disease: A systematic review and meta-analysis of randomized controlled trials. Lipids Health Dis..

[47] Coenzyme q10. https://www.cancer.gov/about-cancer/treatment/cam/hp/coenzyme-q10-pdq.

[48] Vaisar T., Wang S., Omer M., Irwin A.D., Storey C., Tang C., den Hartigh L.J. (2022). 10,12-conjugated linoleic acid supplementation improves hdl composition and function in mice. J. Lipid Res..

[49] Derakhshande-Rishehri S.M., Mansourian M., Kelishadi R., Heidari-Beni M. (2015). Association of foods enriched in conjugated linoleic acid (cla) and cla supplements with lipid profile in human studies: A systematic review and meta-analysis. Public Health Nutr..

[50] Joseph S.V., Jacques H., Plourde M., Mitchell P.L., McLeod R.S., Jones P.J. (2011). Conjugated linoleic acid supplementation for 8 weeks does not affect body composition, lipid profile, or safety biomarkers in overweight, hyperlipidemic men. J. Nutr..

[51] Al M.D., van Houwelingen A.C., Badart-Smook A., Hornstra G. (1995). Some aspects of neonatal essential fatty acid status are altered by linoleic acid supplementation of women during pregnancy. J Nutr.

[52] Kumar P., Malhotra P., Ma K., Singla A., Hedroug O., Saksena S., Dudeja P.K., Gill R.K., Alrefai W.A. (2011). Srebp2 mediates the modulation of intestinal npc1l1 expression by curcumin. Am. J. Physiol. Gastrointest. Liver Physiol..

[53] Tai M.H., Chen P.K., Chen P.Y., Wu M.J., Ho C.T., Yen J.H. (2014). Curcumin enhances cell-surface ldlr level and promotes ldl uptake through downregulation of pcsk9 gene expression in hepg2 cells. Mol. Nutr. Food Res..

[54] Lin X.L., Liu M.H., Hu H.J., Feng H.R., Fan X.J., Zou W.W., Pan Y.Q., Hu X.M., Wang Z. (2015). Curcumin enhanced cholesterol efflux by upregulating abca1 expression through ampk-sirt1-lxralpha signaling in thp-1 macrophage-derived foam cells. DNA Cell Biol..

[55] Sahebkar A. (2014). A systematic review and meta-analysis of randomized controlled trials investigating the effects of curcumin on blood lipid levels. Clin. Nutr..

[56] Tumeric. NIH. https://www.nccih.nih.gov/health/turmeric.

[57] Devaraj R.D., Reddy C.K., Xu B. (2019). Health-promoting effects of konjac glucomannan and its practical applications: A critical review. Int. J. Biol. Macromol..

[58] Ho H.V.T., Jovanovski E., Zurbau A., Blanco Mejia S., Sievenpiper J.L., Au-Yeung F., Jenkins A.L., Duvnjak L., Leiter L., Vuksan V. (2017). A systematic review and meta-analysis of randomized controlled trials of the effect of konjac glucomannan, a viscous soluble fiber, on ldl cholesterol and the new lipid targets non-hdl cholesterol and apolipoprotein b. Am. J. Clin. Nutr..

[59] Henrion M., Francey C., Le K.A., Lamothe L. (2019). Cereal b-glucans: The impact of processing and how it affects physiological responses. Nutrients.

[60] Yu J., Xia J., Yang C., Pan D., Xu D., Sun G., Xia H. (2022). Effects of oat beta-glucan intake on lipid profiles in hypercholesterolemic adults: A systematic review and meta-analysis of randomized controlled trials. Nutrients.

[61] Lin J., Sun Y., Santos H.O., Găman M.A., Bhat L.T., Cui Y. (2021). Effects of guar gum supplementation on the lipid profile: A systematic review and meta-analysis of randomized controlled trials. Nutr. Metab. Cardiovasc. Dis..

[62] Gylling H., Riikonen S., Nikkilä K., Savonius H., Miettinen T.A. (1998). Oral guar gum treatment of intrahepatic cholestasis and pruritus in pregnant women: Effects on serum cholestanol and other non-cholesterol sterols. Eur. J. Clin. Investig..

[63] Jovanovski E., Yashpal S., Komishon A., Zurbau A., Blanco Mejia S., Ho H.V.T., Li D., Sievenpiper J., Duvnjak L., Vuksan V. (2018). Effect of psyllium (*Plantago ovata*) fiber on ldl cholesterol and alternative lipid targets, non-hdl cholesterol and apolipoprotein b: A systematic review and meta-analysis of randomized controlled trials. Am. J. Clin. Nutr..

[64] Chen C., Shang C., Xin L., Xiang M., Wang Y., Shen Z., Jiao L., Ding F., Cui X. (2022). Beneficial effects of psyllium on the prevention and treatment of cardiometabolic diseases. Food Funct..

[65] Shirah B.H., Shirah H.A., Fallata A.H., Alobidy S.N., Al Hawsawi M.M. (2018). Hemorrhoids during pregnancy: Sitz bath vs. Ano-rectal cream: A comparative prospective study of two conservative treatment protocols. Women Birth.

[66] Borlinghaus J., Albrecht F., Gruhlke M.C., Nwachukwu I.D., Slusarenko A.J. (2014). Allicin: Chemistry and biological properties. Molecules.

[67] Sun Y.E., Wang W., Qin J. (2018). Anti-hyperlipidemia of garlic by reducing the level of total cholesterol and low-density lipoprotein: A meta-analysis. Medicine.

[68] Ried K., Toben C., Fakler P. (2013). Effect of garlic on serum lipids: An updated meta-analysis. Nutr. Rev..

[69] Banach M., Patti A.M., Giglio R.V., Cicero A.F.G., Atanasov A.G., Bajraktari G., Bruckert E., Descamps O., Djuric D.M., Ezhov M. (2018). The role of nutraceuticals in statin intolerant patients. J. Am. Coll. Cardiol..

[70] Huang S., Chen H., Teng J., Wu Z.L., Wei L., Xia N. (2022). Antihyperlipidemic effect and increased antioxidant enzyme levels of aqueous extracts from liupao tea and green tea in vivo. J. Food Sci..

[71] Weng X., Odouli R., Li D.K. (2008). Maternal caffeine consumption during pregnancy and the risk of miscarriage: A prospective cohort study. Am. J. Obstet. Gynecol..

[72] Erickson N., Zafron M., Harding S.V., Marinangeli C.P., Rideout T.C. (2020). Evaluating the lipid-lowering effects of alpha-lipoic acid supplementation: A systematic review. J. Diet. Suppl..

[73] Mahmoudinezhad M., Farhangi M.A. (2021). Alpha lipoic acid supplementation affects serum lipids in a dose and duration-dependent manner in different health status. Int. J. Vitam. Nutr. Res..

[74] Najafi N., Mehri S., Ghasemzadeh Rahbardar M., Hosseinzadeh H. (2022). Effects of alpha lipoic acid on metabolic syndrome: A comprehensive review. Phytother. Res..

[75] Lammi C., Fassi E.M.A., Li J., Bartolomei M., Benigno G., Roda G., Arnoldi A., Grazioso G. (2022). Computational design and biological evaluation of analogs of lupin peptide p5 endowed with dual pcsk9/hmg-coar inhibiting activity. Pharmaceutics.

[76] Lammi C., Bollati C., Lecca D., Abbracchio M.P., Arnoldi A. (2019). Lupin peptide t9 (gqeqshqdegvivr) modulates the mutant pcsk9(d374y) pathway: In vitro characterization of its dual hypocholesterolemic behavior. Nutrients.

[77] Lammi C., Zanoni C., Scigliuolo G.M., D’Amato A., Arnoldi A. (2014). Arnoldi. Lupin peptides lower low-density lipoprotein (ldl) cholesterol through an up-regulation of the ldl receptor/sterol regulatory element binding protein 2 (srebp2) pathway at hepg2 cell line. J. Agric. Food Chem..

[78] Bähr M., Fechner A., Krämer J., Kiehntopf M., Jahreis G. (2013). Lupin protein positively affects plasma ldl cholesterol and ldl:Hdl cholesterol ratio in hypercholesterolemic adults after four weeks of supplementation: A randomized, controlled crossover study. Nutr. J..

[79] Zhang Q., Qian Z.Y., Zhou P.H., Zhou X.L., Zhang D.L., He N., Zhang J., Liu Y.H., Gu Q. (2018). Effects of oral selenium and magnesium co-supplementation on lipid metabolism, antioxidative status, histopathological lesions, and related gene expression in rats fed a high-fat diet. Lipids Health Dis..

[80] Asbaghi O., Moradi S., Nezamoleslami S., Moosavian S.P., Hojjati Kermani M.A., Lazaridi A.V., Miraghajani M. (2021). The effects of magnesium supplementation on lipid profile among type 2 diabetes patients: A systematic review and meta-analysis of randomized controlled trials. Biol. Trace Elem. Res..

[81] Simental-Mendia L.E., Simental-Mendia M., Sahebkar A., Rodriguez-Moran M., Guerrero-Romero F. (2017). Effect of magnesium supplementation on lipid profile: A systematic review and meta-analysis of randomized controlled trials. Eur. J. Clin. Pharmacol..

[82] Liu M., Dudley S.C. (2020). Magnesium, oxidative stress, inflammation, and cardiovascular disease. Antioxidants.

[83] Van Laecke S. (2019). Hypomagnesemia and hypermagnesemia. Acta Clin. Belg..

[84] Kamanna V.S., Kashyap M.L. (2008). Mechanism of action of niacin. Am. J. Cardiol..

[85] HPS2-Thrive Collaborative Group (2014). Effects of extended-release niacin with laropiprant in high-risk patients. N. Engl. J. Med..

[86] Aim-High Investigators (2011). Niacin in patients with low hdl cholesterol levels receiving intensive statin therapy. N. Engl. J. Med..

[87] Palawaththa S., Islam R.M., Illic D., Rabel K., Lee M., Romero L., Leung X.Y., Karim M.N. (2022). Effect of maternal dietary niacin intake on congenital anomalies: A systematic review and meta-analysis. Eur. J. Nutr..

[88] Niacin. https://ods.od.nih.gov/factsheets/Niacin-HealthProfessional/#h9.

[89] Asgary S., Sahebkar A., Goli-Malekabadi N. (2015). Ameliorative effects of nigella sativa on dyslipidemia. J. Endocrinol. Investig..

[90] Hallajzadeh J., Milajerdi A., Mobini M., Amirani E., Azizi S., Nikkhah E., Bahadori B., Sheikhsoleimani R., Mirhashemi S.M. (2020). Effects of nigella sativa on glycemic control, lipid profiles, and biomarkers of inflammatory and oxidative stress: A systematic review and meta-analysis of randomized controlled clinical trials. Phytother. Res..

[91] Jahan S., Mozumder Z.M., Shill D.K. (2022). Use of herbal medicines during pregnancy in a group of bangladeshi women. Heliyon.

[92] Lockyer S., Yaqoob P., Spencer J.P.E., Rowland I. (2012). Olive leaf phenolics and cardiovascular risk reduction: Physiological effects and mechanisms of action. Nutr. Aging.

[93] Lockyer S., Rowland I., Spencer J.P.E., Yaqoob P., Stonehouse W. (2017). Impact of phenolic-rich olive leaf extract on blood pressure, plasma lipids and inflammatory markers: A randomised controlled trial. Eur. J. Nutr..

[94] Olive. https://medlineplus.gov/druginfo/natural/233.html.

[95] Ramazani E., Akaberi M., Emami S.A., Tayarani-Najaran Z. (2021). Biological and pharmacological effects of gamma-oryzanol: An updated review of the molecular mechanisms. Curr. Pharm. Des..

[96] Yan S., Chen J., Zhu L., Guo T., Qin D., Hu Z., Han S., Zhou Y., Akan O.D., Wang J. (2022). Oryzanol attenuates high fat and cholesterol diet-induced hyperlipidemia by regulating the gut microbiome and amino acid metabolism. J. Agric. Food Chem..

[97] Pourrajab B., Sohouli M.H., Amirinejad A., Fatahi S., Gaman M.A., Shidfar F. (2022). The impact of rice bran oil consumption on the serum lipid profile in adults: A systematic review and meta-analysis of randomized controlled trials. Crit. Rev. Food Sci. Nutr..

[98] Evans M., Rumberger J.A., Azumano I., Napolitano J.J., Citrolo D., Kamiya T. (2014). Pantethine, a derivative of vitamin b5, favorably alters total, ldl and non-hdl cholesterol in low to moderate cardiovascular risk subjects eligible for statin therapy: A triple-blinded placebo and diet-controlled investigation. Vasc. Health Risk Manag..

[99] Donati C., Barbi G., Cairo G., Prati G.F., Degli Esposti E. (1986). Pantethine improves the lipid abnormalities of chronic hemodialysis patients: Results of a multicenter clinical trial. Clin. Nephrol..

[100] Bertolini S., Donati C., Elicio N., Daga A., Cuzzolaro S., Marcenaro A., Saturnino M., Balestreri R. (1986). Lipoprotein changes induced by pantethine in hyperlipoproteinemic patients: Adults and children. Int. J. Clin. Pharmacol. Ther. Toxicol..

[101] Harris W.S., Bulchandani S. (2006). Why do omega-3 fatty acids lower serum triglycerides?. Curr. Opin. Lipidol..

[102] EFSA Panel on Dietetic Products, Nutrition and Allergies (2009). Scientific opinion on the substantiation of health claims related to epa, dha, dpa and maintenance of normal blood pressure (id 502), maintenance of normal hdl-cholesterol concentrations (id 515), maintenance of normal (fasting) blood concentrations of triglycerides (ID 517), maintenance of normal LDL-cholesterol concentrations (ID 528, 698) and maintenance of joints (ID 503, 505, 507, 511, 518, 524, 526, 535, 537) pursuant to Article 13(1) of Regulation (EC) No 1924/2006. EFSA J..

[103] Nicholls S.J., Lincoff A.M., Garcia M., Bash D., Ballantyne C.M., Barter P.J., Davidson M.H., Kastelein J.J.P., Koenig W., McGuire D.K. (2020). Effect of high-dose omega-3 fatty acids vs corn oil on major adverse cardiovascular events in patients at high cardiovascular risk: The strength randomized clinical trial. JAMA.

[104] Zhang H.J., Gao X., Guo X.F., Li K.L., Li S., Sinclair A.J., Li D. (2021). Effects of dietary eicosapentaenoic acid and docosahexaenoic acid supplementation on metabolic syndrome: A systematic review and meta-analysis of data from 33 randomized controlled trials. Clin. Nutr..

[105] Bhatt D.L., Steg P.G., Miller M., Brinton E.A., Jacobson T.A., Ketchum S.B., Doyle R.T., Juliano R.A., Jiao L., Granowitz C. (2019). Cardiovascular risk reduction with icosapent ethyl for hypertriglyceridemia. N. Engl. J. Med..

[106] Saha S., Saha S. (2022). The effects of prenatal dietary supplements on blood glucose and lipid metabolism in gestational diabetes mellitus patients: A systematic review and network meta-analysis protocol of randomized controlled trials. PLoS ONE.

[107] Brouns F., Theuwissen E., Adam A., Bell M., Berger A., Mensink R.P. (2012). Cholesterol-lowering properties of different pectin types in mildly hyper-cholesterolemic men and women. Eur. J. Clin. Nutr..

[108] Hu H., Zhang S., Liu F., Zhang P., Muhammad Z., Pan S. (2019). Role of the gut microbiota and their metabolites in modulating the cholesterol-lowering effects of citrus pectin oligosaccharides in c57bl/6 mice. J. Agric. Food Chem..

[109] (1991). Fermentable fibers and vitamin b12 dependency. Nutr. Rev..

[110] Shaghaghi M.A., Abumweis S.S., Jones P.J. (2013). Cholesterol-lowering efficacy of plant sterols/stanols provided in capsule and tablet formats: Results of a systematic review and meta-analysis. J. Acad. Nutr. Diet..

[111] Sabeva N.S., McPhaul C.M., Li X., Cory T.J., Feola D.J., Graf G.A. (2011). Phytosterols differentially influence abc transporter expression, cholesterol efflux and inflammatory cytokine secretion in macrophage foam cells. J. Nutr. Biochem..

[112] Ho S.S., Pal S. (2005). Margarine phytosterols decrease the secretion of atherogenic lipoproteins from hepg2 liver and caco2 intestinal cells. Atherosclerosis.

[113] Gao F., Wang G., Wang L., Guo N. (2017). Phytosterol nutritional supplement improves pregnancy and neonatal complications of gestational diabetes mellitus in a double-blind and placebo-controlled clinical study. Food Funct..

[114] Singh D.K., Li L., Porter T.D. (2006). Policosanol inhibits cholesterol synthesis in hepatoma cells by activation of amp-kinase. J. Pharmacol Exp. Ther..

[115] Zhai Z., Niu K.M., Liu H., Lin C., Tu Y., Liu Y., Cai L., Ouyang K., Liu J. (2021). Policosanol alleviates hepatic lipid accumulation by regulating bile acids metabolism in c57bl6/mice through ampk-fxr-tgr5 cross-talk. J. Food Sci..

[116] Gong J., Qin X., Yuan F., Hu M., Chen G., Fang K., Wang D., Jiang S., Li J., Zhao Y. (2018). Efficacy and safety of sugarcane policosanol on dyslipidemia: A meta-analysis of randomized controlled trials. Mol. Nutr. Food Res..

[117] Rodriguez M.D., Garcia H. (1998). Evaluation of peri- and post-natal toxicity of policosanol in rats. Teratog. Carcinog. Mutagen..

[118] Rodríguez M.D., García H. (1994). Teratogenic and reproductive studies of policosanol in the rat and rabbit. Teratog. Carcinog. Mutagen..

[119] Kim G.B., Yi S.H., Lee B.H. (2004). Purification and characterization of three different types of bile salt hydrolases from bifidobacterium strains. J. Dairy Sci..

[120] Yoon H., Lee Y., Kang H.J., Ju J., Ji Y., Park H., Lee H., Holzapfel W.H. (2022). Two putative probiotic strains improve diet-induced hypercholesterolemia through modulating intestinal cholesterol uptake and hepatic cholesterol efflux. J. Appl. Microbiol..

[121] Yang M., Zheng J., Zong X., Yang X., Zhang Y., Man C., Jiang Y. (2021). Preventive effect and molecular mechanism of lactobacillus rhamnosus jl1 on food-borne obesity in mice. Nutrients.

[122] Sun K., Liu Z., Wang H. (2022). The effect of probiotics on the serum lipid levels in non-obese healthy adults with hyperlipidemia: A systematic review and meta-analysis of randomized controlled trials. Nutr. Hosp..

[123] Probiotics Fact Sheet for Health Professionals. https://ods.od.nih.gov/factsheets/Probiotics-HealthProfessional/.

[124] Sirtori C.R. (2014). The pharmacology of statins. Pharmacol. Res..

[125] (2021). Statins: Drug Safety Communication–FDA Requests Removal of Strongest Warning Against Using Cholesterol-Lowering Statins during Pregnancy.

[126] Banach M., Bruckert E., Descamps O.S., Ellegard L., Ezhov M., Foger B., Fras Z., Kovanen P.T., Latkovskis G., Marz W. (2019). The role of red yeast rice (ryr) supplementation in plasma cholesterol control: A review and expert opinion. Atheroscler. Suppl..

[127] Ahn J., Cho I., Kim S., Kwon D., Ha T. (2008). Dietary resveratrol alters lipid metabolism-related gene expression of mice on an atherogenic diet. J. Hepatol..

[128] Dong W., Wang X., Bi S., Pan Z., Liu S., Yu H., Lu H., Lin X., Wang X., Ma T. (2014). Inhibitory effects of resveratrol on foam cell formation are mediated through monocyte chemotactic protein-1 and lipid metabolism-related proteins. Int. J. Mol. Med..

[129] Resveratrol. https://medlineplus.gov/druginfo/natural/307.html.

[130] Simental-Mendia L.E., Guerrero-Romero F. (2019). Effect of resveratrol supplementation on lipid profile in subjects with dyslipidemia: A randomized double-blind, placebo-controlled trial. Nutrition.

[131] Cao X., Liao W., Xia H., Wang S., Sun G. (2022). The effect of resveratrol on blood lipid profile: A dose-response meta-analysis of randomized controlled trials. Nutrients.

[132] Xiao P.T., Liu S.Y., Kuang Y.J., Jiang Z.M., Lin Y., Xie Z.S., Liu E.H. (2022). Network pharmacology analysis and experimental validation to explore the mechanism of sea buckthorn flavonoids on hyperlipidemia. J. Ethnopharmacol..

[133] Geng Y., Wang J., Chen K., Li Q., Ping Z., Xue R., Zhang S. (2022). Effects of sea buckthorn (*Hippophae rhamnoides* L.) on factors related to metabolic syndrome: A systematic review and meta-analysis of randomized controlled trial. Phytother. Res..

[134] Wen P., Zhao P., Qin G., Tang S., Li B., Zhang J., Peng L. (2020). Genotoxicity and teratogenicity of seabuckthorn (*Hippophae rhamnoides* L.) berry oil. Drug Chem. Toxicol..

[135] Xiao P., Ji H., Ye Y., Zhang B., Chen Y., Tian J., Liu P., Chen L., Du Z. (2017). Dietary silymarin supplementation promotes growth performance and improves lipid metabolism and health status in grass carp (*Ctenopharyngodon idellus*) fed diets with elevated lipid levels. Fish Physiol. Biochem..

[136] Wang L., Rotter S., Ladurner A., Heiss E.H., Oberlies N.H., Dirsch V.M., Atanasov A.G. (2015). Silymarin constituents enhance abca1 expression in thp-1 macrophages. Molecules.

[137] Soleymani S., Ayati M.H., Mansourzadeh M.J., Namazi N., Zargaran A. (2022). The effects of silymarin on the features of cardiometabolic syndrome in adults: A systematic review and meta-analysis. Phytother. Res..

[138] (2022). Milk Thistle. https://www.nccih.nih.gov/health/milk-thistle.

[139] Li T.T., Tong A.J., Liu Y.Y., Huang Z.R., Wan X.Z., Pan Y.Y., Jia R.B., Liu B., Chen X.H., Zhao C. (2019). Polyunsaturated fatty acids from microalgae spirulina platensis modulates lipid metabolism disorders and gut microbiota in high-fat diet rats. Food Chem. Toxicol..

[140] Hatami E., Ghalishourani S.S., Najafgholizadeh A., Pourmasoumi M., Hadi A., Clark C.C., Assaroudi M., Salehi-Sahlabadi A., Joukar F., Mansour-Ghanaei F. (2021). The effect of spirulina on type 2 diabetes: A systematic review and meta-analysis. J. Diabetes Metab. Disord..

[141] Macchi C., Greco M.F., Ferri N., Magni P., Arnoldi A., Corsini A., Sirtori C.R., Ruscica M., Lammi C. (2021). Impact of soy beta-conglycinin peptides on pcsk9 protein expression in hepg2 cells. Nutrients.

[142] Cho S.J., Juillerat M.A., Lee C.H. (2007). Cholesterol lowering mechanism of soybean protein hydrolysate. J. Agric. Food Chem..

[143] Potter S.M. (1995). Overview of proposed mechanisms for the hypocholesterolemic effect of soy. J. Nutr..

[144] Butteiger D.N., Hibberd A.A., McGraw N.J., Napawan N., Hall-Porter J.M., Krul E.S. (2016). Soy protein compared with milk protein in a western diet increases gut microbial diversity and reduces serum lipids in golden syrian hamsters. J. Nutr..

[145] Napoli C., Leccese M., Palumbo G., De Nigris F., Chiariello P., Zuliani P., Somma P., Di Loreto M., De Matteis C., Cacciatore F. (1998). Effects of vitamin e and hmg-coa reductase inhibition on cholesteryl ester transfer protein and lecithin-cholesterol acyltransferase in hypercholesterolemia. Coron. Artery Dis..

[146] Li F., Tan W., Kang Z., Wong C.W. (2010). Tocotrienol enriched palm oil prevents atherosclerosis through modulating the activities of peroxisome proliferators-activated receptors. Atherosclerosis.

[147] Traber M.G., Head B. (2021). Vitamin e: How much is enough, too much and why!. Free Radic. Biol. Med..

[148] Zuo S., Wang G., Han Q., Xiao H., Santos H.O., Rodriguez D.A., Khani V., Tang J. (2020). The effects of tocotrienol supplementation on lipid profile: A meta-analysis of randomized controlled trials. Complement Ther. Med..

[149] Saremi A., Arora R. (2010). Vitamin e and cardiovascular disease. Am. J. Ther..

[150] Yuan R., Yuan Y., Wang L., Xin Q., Wang Y., Shi W., Miao Y., Leng S.X., Chen K., Cong W. (2022). Red yeast rice preparations reduce mortality, major cardiovascular adverse events, and risk factors for metabolic syndrome: A systematic review and meta-analysis. Front. Pharmacol..

[151] Li P., Wang Q., Chen K., Zou S., Shu S., Lu C., Wang S., Jiang Y., Fan C., Luo Y. (2021). Red yeast rice for hyperlipidemia: A meta-analysis of 15 high-quality randomized controlled trials. Front. Pharmacol..

[152] Sungthong B., Yoothaekool C., Promphamorn S., Phimarn W. (2020). Efficacy of red yeast rice extract on myocardial infarction patients with borderline hypercholesterolemia: A meta-analysis of randomized controlled trials. Sci. Rep..

[153] Younes M., Aggett P., Aguilar F., Crebelli R., Dusemund B., Filipič M., Frutos M.J., Galtier P., Gott D., Gundert-Remy U. (2018). Scientific opinion on the safety of monacolins in red yeast rice. EFSA J..

[154] Visseren F.L.J., Mach F., Smulders Y.M., Carballo D., Koskinas K.C., Bäck M., Benetos A., Biffi A., Boavida J.M., Capodanno D. (2022). 2021 esc guidelines on cardiovascular disease prevention in clinical practice. Eur. J. Prev. Cardiol..

[155] Sahebkar A., Serban C., Ursoniu S., Banach M. (2016). Effect of garlic on plasma lipoprotein(a) concentrations: A systematic review and meta-analysis of randomized controlled clinical trials. Nutrition.

[156] Matsumoto S., Nakanishi R., Li D., Alani A., Rezaeian P., Prabhu S., Abraham J., Fahmy M.A., Dailing C., Flores F. (2016). Aged garlic extract reduces low attenuation plaque in coronary arteries of patients with metabolic syndrome in a prospective randomized double-blind study. J. Nutr..

[157] Shaikh K., Kinninger A., Cherukuri L., Birudaraju D., Nakanishi R., Almeida S., Jayawardena E., Shekar C., Flores F., Hamal S. (2020). Aged garlic extract reduces low attenuation plaque in coronary arteries of patients with diabetes: A randomized, double-blind, placebo-controlled study. Exp. Ther. Med..

[158] Ben Salem M., Affes H., Ksouda K., Dhouibi R., Sahnoun Z., Hammami S., Zeghal K.M. (2015). Pharmacological studies of artichoke leaf extract and their health benefits. Plant Foods Hum. Nutr..

[159] Henning S.M., Fajardo-Lira C., Lee H.W., Youssefian A.A., Go V.L., Heber D. (2003). Catechin content of 18 teas and a green tea extract supplement correlates with the antioxidant capacity. Nutr. Cancer.

[160] Busnelli M., Manzini S., Sirtori C.R., Chiesa G., Parolini C. (2018). Effects of vegetable proteins on hypercholesterolemia and gut microbiota modulation. Nutrients.

[161] Blanco Mejia S., Messina M., Li S.S., Viguiliouk E., Chiavaroli L., Khan T.A., Srichaikul K., Mirrahimi A., Sievenpiper J.L., Kris-Etherton P. (2019). A meta-analysis of 46 studies identified by the fda demonstrates that soy protein decreases circulating ldl and total cholesterol concentrations in adults. J. Nutr..

[162] Simental-Mendia L.E., Gotto A.M., Atkin S.L., Banach M., Pirro M., Sahebkar A. (2018). Effect of soy isoflavone supplementation on plasma lipoprotein(a) concentrations: A meta-analysis. J. Clin. Lipidol..

[163] Ying J., Zhang Y., Yu K. (2019). Phytosterol compositions of enriched products influence their cholesterol-lowering efficacy: A meta-analysis of randomized controlled trials. Eur. J. Clin. Nutr..

[164] Jacobson T.A., Maki K.C., Orringer C., Jones E.P., Kris-Etherton H.P., Sikand G., La Forge R., Daniels S.R., Wilson D.P., Morris P.B. (2015). National lipid association recommendations for patient-centered management of dyslipidemia: Part 2. J. Clin. Lipidol..

[165] EFSA Panel on Dietetic Products, Nutrition and Allergies (2015). Scientific opinion on the substantiation of health claims related to dietary fibre and maintenance of normal blood cholesterol concentrations (id 747, 750, 811) pursuant to article 13(1) of regulation (ec) no 1924/2006. EFSA J..

[166] Soltani M.D., Meftahizadeh H., Barani M., Rahdar A., Hosseinikhah S.M., Hatami M., Ghorbanpour M. (2021). Guar (*Cyamopsis tetragonoloba* L.) plant gum: From biological applications to advanced nanomedicine. Int. J. Biol. Macromol..

[167] Packer L., Witt E.H., Tritschler H.J. (1995). Alpha-lipoic acid as a biological antioxidant. Free Radic. Biol. Med..

[168] Budoff M.J., Bhatt D.L., Kinninger A., Lakshmanan S., Muhlestein J.B., Le V.T., May H.T., Shaikh K., Shekar C., Roy S.K. (2020). Effect of icosapent ethyl on progression of coronary atherosclerosis in patients with elevated triglycerides on statin therapy: Final results of the evaporate trial. Eur. Heart J..

[169] Xiao Y., Zhang Q., Liao X., Elbelt U., Weylandt K.H. (2022). The effects of omega-3 fatty acids in type 2 diabetes: A systematic review and meta-analysis. Prostaglandins Leukot. Essent. Fat. Acids.

[170] Saito Y., Yokoyama M., Origasa H., Matsuzaki M., Matsuzawa Y., Ishikawa Y., Oikawa S., Sasaki J., Hishida H., Itakura H. (2008). Effects of epa on coronary artery disease in hypercholesterolemic patients with multiple risk factors: Sub-analysis of primary prevention cases from the japan epa lipid intervention study (jelis). Atherosclerosis.

[171] D’Andrea E., Hey S.P., Ramirez C.L., Kesselheim A.S. (2019). Assessment of the role of niacin in managing cardiovascular disease outcomes: A systematic review and meta-analysis. JAMA Netw. Open.

[172] Sahebkar A., Reiner Ž., Simental-Mendia L.E., Ferretti G., Cicero A.F. (2016). Effect of extended-release niacin on plasma lipoprotein(a) levels: A systematic review and meta-analysis of randomized placebo-controlled trials. Metabolism.

[173] Food and Drug Administration (2016). Withdrawal of approval of indications related to the coadministration with statins in applications for niacin extended-release tablets and fenofibric acid delayed-release capsules. Fed. Regist..

[174] (2013). European Medicines Agency Confirms Recommendation to Suspend Tredaptive, Pelzont and Trevaclyn. https://www.ema.europa.eu/en/news/european-medicines-agency-confirms-recommendation-suspend-tredaptive-pelzont-trevaclyn.

[175] DiNicolantonio J.J. (2012). Coq10 and l-carnitine for statin myalgia?. Expert. Rev. Cardiovasc. Ther..

[176] Serban M.C., Sahebkar A., Mikhailidis D.P., Toth P.P., Jones S.R., Muntner P., Blaha M.J., Andrica F., Martin S.S., Borza C. (2016). Impact of l-carnitine on plasma lipoprotein(a) concentrations: A systematic review and meta-analysis of randomized controlled trials. Sci. Rep..

[177] Salonen R.M., Nyyssönen K., Kaikkonen J., Porkkala-Sarataho E., Voutilainen S., Rissanen T.H., Tuomainen T.P., Valkonen V.P., Ristonmaa U., Lakka H.M. (2003). Six-year effect of combined vitamin c and e supplementation on atherosclerotic progression: The antioxidant supplementation in atherosclerosis prevention (asap) study. Circulation.

[178] Akbari M., Tamtaji O.R., Lankarani K.B., Tabrizi R., Dadgostar E., Haghighat N., Kolahdooz F., Ghaderi A., Mansournia M.A., Asemi Z. (2020). The effects of resveratrol on lipid profiles and liver enzymes in patients with metabolic syndrome and related disorders: A systematic review and meta-analysis of randomized controlled trials. Lipids Health Dis..

[179] Khalili L., Nammi S. (2022). The effects of curcumin supplementation on metabolic biomarkers and body mass index in patients with nonalcoholic fatty liver disease: A systematic review and meta-analysis of randomized controlled trials. Curr. Pharmacol. Des..

[180] Altobelli E., Angeletti P.M., Marziliano C., Mastrodomenico M., Giuliani A.R., Petrocelli R. (2021). Potential therapeutic effects of curcumin on glycemic and lipid profile in uncomplicated type 2 diabetes-a meta-analysis of randomized controlled trial. Nutrients.

[181] Mahdi G.S. (1996). Chromium deficiency might contribute to insulin resistance, type 2 diabetes mellitus, dyslipidaemia, and atherosclerosis. Diabet. Med..

[182] Warden B.A., Guyton J.R., Kovacs A.C., Durham J.A., Jones L.K., Dixon D.L., Jacobson T.A., Duell P.B. (2022). Assessment and management of statin-associated muscle symptoms (sams): A clinical perspective from the national lipid association. J. Clin. Lipidol..

[183] Alehagen U., Aaseth J., Alexander J., Johansson P. (2018). Still reduced cardiovascular mortality 12 years after supplementation with selenium and coenzyme q10 for four years: A validation of previous 10-year follow-up results of a prospective randomized double-blind placebo-controlled trial in elderly. PLoS ONE.

[184] Alehagen U., Johansson P., Björnstedt M., Rosén A., Dahlström U. (2013). Cardiovascular mortality and n-terminal-probnp reduced after combined selenium and coenzyme q10 supplementation: A 5-year prospective randomized double-blind placebo-controlled trial among elderly swedish citizens. Int. J. Cardiol..

[185] AHA eStaff (2020). What is the Mediterranean Diet?. https://www.heart.org/en/healthy-living/healthy-eating/eat-smart/nutrition-basics/mediterranean-diet#:~:text=A%20Mediterranean-style%20diet%20typically%20includes%3A%201%20plenty%20of,fish%20and%20poultry%20in%20low%20to%20moderate%20amounts.

[186] Arnett D.K., Blumenthal R.S., Albert M.A., Buroker A.B., Goldberger Z.D., Hahn E.J., Himmelfarb C.D., Khera A., Lloyd-Jones D., McEvoy J.W. (2019). 2019 acc/aha guideline on the primary prevention of cardiovascular disease: A report of the american college of cardiology/american heart association task force on clinical practice guidelines. Circulation.

[187] Papadaki A., Nolen-Doerr E., Mantzoros C.S. (2020). The effect of the mediterranean diet on metabolic health: A systematic review and meta-analysis of controlled trials in adults. Nutrients.

[188] Estruch R., Ros E., Salas-Salvadó J., Covas M.I., Corella D., Arós F., Gómez-Gracia E., Ruiz-Gutiérrez V., Fiol M., Lapetra J. (2018). Primary prevention of cardiovascular disease with a mediterranean diet supplemented with extra-virgin olive oil or nuts. N. Engl. J. Med..

[189] Martínez-González M.A., Sánchez-Tainta A., Corella D., Salas-Salvado J., Ros E., Arós F., Gómez-Gracia E., Fiol M., Lamuela-Raventós R.M., Schröder H. (2014). A provegetarian food pattern and reduction in total mortality in the prevención con dieta mediterránea (predimed) study. Am. J. Clin. Nutr..

[190] Tang C., Wang X., Qin L.Q., Dong J.Y. (2021). Mediterranean diet and mortality in people with cardiovascular disease: A meta-analysis of prospective cohort studies. Nutrients.

[191] Leucht S., Chaimani A., Cipriani A.S., Davis J.M., Furukawa T.A., Salanti G. (2016). Network meta-analyses should be the highest level of evidence in treatment guidelines. Eur. Arch. Psychiatry Clin. Neurosci..

[192] Laffin L.J., Bruemmer D., Garcia M., Brennan D.M., McErlean E., Jacoby D.S., Michos E.D., Ridker P.M., Wang T.Y., Watson K.E. (2022). Comparative effects of low-dose rosuvastatin, placebo and dietary supplements on lipids and inflammatory biomarkers. J. Am. Coll. Cardiol..

